# A Cautionary Note on Predicting Social Judgments from Faces with Deep Neural Networks

**DOI:** 10.1007/s42761-021-00075-5

**Published:** 2021-09-20

**Authors:** Umit Keles, Chujun Lin, Ralph Adolphs

**Affiliations:** 1 Division of the Humanities and Social Sciences, California Institute of Technology, Pasadena, CA USA; 2 Department of Psychological and Brain Sciences, Dartmouth College, Hanover, NH USA; 3 Division of Biology and Biological Engineering, California Institute of Technology, Pasadena, CA USA

**Keywords:** Social cognition, Face perception, Traits, Affective states, Deep neural networks

## Abstract

People spontaneously infer other people’s psychology from faces, encompassing inferences of their affective states, cognitive states, and stable traits such as personality. These judgments are known to be often invalid, but nonetheless bias many social decisions. Their importance and ubiquity have made them popular targets for automated prediction using deep convolutional neural networks (DCNNs). Here, we investigated the applicability of this approach: how well does it generalize, and what biases does it introduce? We compared three distinct sets of features (from a face identification DCNN, an object recognition DCNN, and using facial geometry), and tested their prediction across multiple out-of-sample datasets. Across judgments and datasets, features from both pre-trained DCNNs provided better predictions than did facial geometry. However, predictions using object recognition DCNN features were not robust to superficial cues (e.g., color and hair style). Importantly, predictions using face identification DCNN features were not specific: models trained to predict one social judgment (e.g., trustworthiness) also significantly predicted other social judgments (e.g., femininity and criminal), and at an even higher accuracy in some cases than predicting the judgment of interest (e.g., trustworthiness). Models trained to predict affective states (e.g., happy) also significantly predicted judgments of stable traits (e.g., sociable), and vice versa. Our analysis pipeline not only provides a flexible and efficient framework for predicting affective and social judgments from faces but also highlights the dangers of such automated predictions: correlated but unintended judgments can drive the predictions of the intended judgments.

**Supplementary Information:**

The online version contains supplementary material available at 10.1007/s42761-021-00075-5.

## Introduction

People rapidly and spontaneously make judgments about unfamiliar others’ social attributes based on their faces, such as forming an impression that someone looks beautiful, trustworthy, or happy (Engell et al., 2007; Sutherland et al., 2018; Willis & Todorov, 2006). By and large, these judgments are either known to be invalid or are of unknown validity, since the ground truth of how people really feel and what personality they have is generally impossible to infer merely from looking at their faces (Todorov, 2017). Yet these social judgments have ubiquitous and major consequences in everyday life. For instance, a large body of research has demonstrated that social judgments of political candidates based merely on faces (e.g., how competent an unfamiliar candidate looks) are associated with election outcomes across various regions of the world (Lin et al., 2017; Martin, 1978; Todorov et al., 2005). Some evidence even suggests that these social judgments from faces causally influence individual voting decisions (Ahler et al., 2017; Lenz & Lawson, 2011). Other examples of social judgments from faces influencing real-life decisions range from picking out dates to hiring employees, choosing science news, and determining courtroom sentences (Gheorghiu et al., 2017; Hamermesh, 2011; Oliviola et al., 2015; Wilson & Rule, 2015). In the real world, these judgments can show large individual differences and context effects: not only are they invalid, but consensus can also be difficult to achieve even for stimuli argued to be universal, such as emotional facial expressions (Barrett et al., 2019). With these constraints in mind, it remains a fact that people make many judgments about other people solely from faces in the absence of context or other information (e.g., deciding not to date someone just based on profile photos on dating sites), and the underlying psychological dimensions that explain the most variance in these judgments show considerable consensus across cultures (Lin et al., 2021).

An important applied question is whether machines could be trained to make social judgments from faces like humans do. Recent work has trained deep convolutional neural networks (DCNNs) on face images that had been previously rated on various social attributes to predict how humans would judge new face images on the same set of social attributes (Lewenberg et al., 2017; McCurrie et al., 2018). While this approach is informative, it is difficult to obtain sufficiently dense ratings for training — and turns out to be unnecessary. DCNNs that have only been trained to recognize face identity, or even object identity, without any training specifically on social judgments, already generate features that can be used in linear regression models to predict the social judgments that humans make from faces (Parde et al., 2019; Song et al., 2017). This successful prediction is likely due to the fact that in the absence of any other context, the structural features of the face are also the only source of information that human raters have available for their social judgments. This approach in principle offers a more flexible and scalable framework for practical application: new faces can be projected into the same, pre-trained DCNN to generate facial features, which could then be used in regression models to predict social judgments. This takes advantage of the power of existing pre-trained DCNNs that typically generalize over pose, viewpoint, and image quality, and obviates the need to train new DCNNs or retrain existing networks on domain-specific social judgments, which is inefficient (Hill et al., 2019; O’Toole et al., 2018).

However, past work highlights several specific limitations of using pre-trained DCNNs to predict social judgments that humans make from faces. First, inconsistent results have been found when comparing performance between models using features from DCNNs pre-trained for face identification and those using features from DCNNs pre-trained for object recognition (Parde et al., 2019; Song et al., 2017). It is also unclear to what extent features from different pre-trained DCNNs explain the same or unique variance in social judgments from faces. Second, prior studies trained and tested their models using a single dataset, such as the 10 k US Adult Face Database (Bainbridge et al., 2013) in Song et al. (2017), and the Human ID Database (O’Toole et al., 2005) in Parde et al. (2019). It remains an open question how well this approach generalizes out-of-sample, both across face databases and across human raters, which is a growing concern in modern machine learning for practical applications (D’Amour et al., 2020). Third, recent findings show that social judgments from faces made by human participants on a large number of social attributes can be captured by only a small number (two to four) of psychological dimensions (Lin et al., 2021; Oosterhof & Todorov, 2008; Sutherland et al., 2013). These findings suggest that many social judgments from faces are highly correlated, raising the possibility that models trained to predict one social judgment may also predict other social judgments. If that is the case, then it will be difficult to assert whether models trained to predict one social judgment (e.g., whether someone looks like a criminal) indeed learn the representation of this specific social attribute from faces (criminal) or they instead learn the representation of other social attributes from faces that happen to be stereotypically linked to the perception of criminality (e.g., whether someone looks feminine or masculine; Oldmeadow et al., 2013). Examining this question will offer critical insights into how one should interpret the results from the increasingly popular automated predictions of various social attributes from faces (Bowyer et al., 2020; Wang & Kosinski, 2018).

**Fig. 1:**
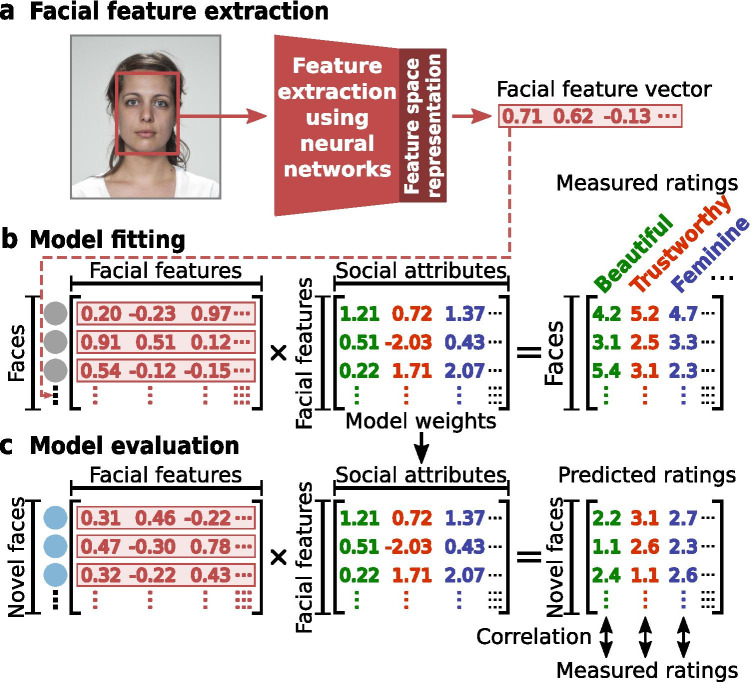
**Overview of modeling framework**. **a** Face images were projected into three distinct feature spaces: a feature space obtained from the top layer of a pre-trained DCNN for face identification (DCNN-Identity; King, 2009, 2017); a feature space obtained from the block5_conv2 layer (Song et al., 2017) of a pre-trained DCNN for object recognition (DCNN-Object; Simonyan & Zisserman, 2015), and a feature space obtained from physical and geometric measures of the faces (Facial-Geometry; Ma et al., 2015). **b** Regularized linear regression with cross-validation was used to estimate a set of model weights for each social attribute, which maps each feature space onto the social judgment ratings measured from human participants. **c** The estimated model weights were then used to predict the measured ratings for novel faces from their facial features. Models constructed for the three distinct feature spaces were compared based on how accurately they predicted ratings for novel faces (Spearman’s correlation between the predicted ratings from the model and the actual ratings collected from human participants)

We address the above three open questions in the present study. We fit regularized linear regression models with cross-validation to predict social judgments from faces made by humans using features from three distinct spaces (Fig. 1a–b; see also “2”): a pre-trained DCNN for face identification (*DCNN-Identity*; King, 2009, 2017), a pre-trained DCNN for object recognition (*DCNN-Object*; Simonyan & Zisserman, 2015), and facial geometry (*Facial-Geometry*; e.g., eye size; see Fig. S2; Ma et al., 2015) for comparison to previous findings (e.g., faces with wider eyes are perceived as more honest; Zebrowitz et al., 1996). All linear regression models were fitted to the neutral, frontal, white faces (*N* = 183) and their corresponding available human subject ratings from the Chicago Face Database (Ma et al., 2015), a widely used database in machine learning studies of faces. To characterize the generalizability of the current approach across faces, raters, and social judgments, we tested the models in five out-of-sample datasets that included ratings for different types of face images on a variety of social attributes provided by independent samples of human subjects (Fig. 1c). To compare the performance across the three distinct feature spaces, we conducted variance partitioning analysis to characterize the shared and unique variance in the social judgments that could be explained by these feature spaces. We also digitally manipulated several aspects of the face images (e.g., color and hair style) and compared how robustly these different feature spaces predicted social judgments from the manipulated faces. Finally, to understand the specificity of these predictions, we examined the cross-predictions across social attributes — that is, how one model trained on a specific social judgment predicted other social judgments. We further investigated the underlying mechanism of these cross-predictions using semi-partial correlation analyses, which sheds light on how different social attributes play a role in leading to cross-predictions.

## Methods

### Training and Test Datasets

The data used in the present research were from publicly available datasets and previously published studies. The linear regression models were fit to 183 studio portraits of neutral, frontal, white faces of men and women and their ratings on various social attributes from the Chicago Face Database (Ma et al., 2015). This database originally contained social attribute ratings for 597 portraits of neutral, frontal faces from four races (Asian, Black, Latino, and White); the other 414 of the 597 faces that are not white were excluded since the effect of race is beyond the scope of our current research. The database provides, for each face, ratings by human subjects on 15 social attributes (*afraid*, *angry*, *attractive*, *baby-faced*, *disgusted*, *dominant*, *feminine*, *happy*, *masculine*, *prototypic*, *sad*, *surprised*, *threatening*, *trustworthy*, and *unusual*) using a 1–7 Likert scale (1 = Not at all, 7 = Extremely). We excluded judgments of *unusual* because neither this social attribute nor its synonym or antonym was rated in any of the out-of-sample test datasets that we used. Thus, we fit 14 linear regression models, one for each of the remaining 14 social attributes. The design matrix for the linear regression had 183 rows. Each row represented one of the 183 face images in the training dataset and each column represented one of the features in the respective facial feature space that was considered (Fig. 1b).

The models were tested on five out-of-sample independent datasets that are publicly available (Lin et al., 2021; Oh et al., 2020; Oosterhof & Todorov, 2008; Walker et al., 2018; White et al., 2017). These test datasets were selected to sample social judgments from different types of faces, including studio portraits of frontal, neutral faces, computer-generated faces, and ambient photos of faces taken under unconstrained conditions. All faces in our training and test datasets were limited to white faces; the effects of race and context (e.g., image background and facial expression) are beyond the scope of our current study. Specifically, the Lin et al. (2021) dataset included ratings for 100 studio portraits of frontal, neutral, white faces (of which 60 were non-overlapping with the training dataset, i.e., 60 novel faces) on 100 social attributes. The Oh et al. (2020) dataset included ratings for 66 novel studio portraits of frontal, neutral, white faces on 14 social attributes. The Walker et al. (2018) dataset included ratings for 40 novel studio portraits of frontal, neutral, white faces on seven social attributes. The Oosterhof and Todorov (2008) dataset included ratings for 300 computer-generated frontal, neutral, white faces on nine social attributes. The White et al. (2017) dataset originally included ratings for 1224 ambient photos (12 images of each of the 102 individuals of various races) taken in real-world contexts downloaded from their Facebook accounts (varied in viewpoint, facial expression, background, illumination, etc.) on five social attributes. We only used 504 photos of white individuals (12 images of each of the 42 individuals). Model training and testing were performed using ratings averaged across human subjects per face per social attribute.

To assess how well the linear regression models with different feature sets predicted social judgment from faces (see the first section of Results, “Generalizability Across Faces, Raters, and Social Attributes”), we fit a model for a social attribute on the Chicago Face Database and tested the model for the same or highly (dis)similar (synonyms/antonyms) social attribute on the out-of-sample test datasets. Ideally, we would fit a model for a social attribute and test the model for the same social attribute. However, the different test datasets generally measured judgments of different social attributes than the training dataset. Therefore, in the case where the same social attribute in the training dataset was not available in the test dataset, we used the synonym/antonym of the fitted social attribute in the test dataset (if available). Based on this rationale, we tested the models that were fit to the corresponding social attributes in the Chicago Face Database on nine social attributes in the Lin et al. (2021)﻿ dataset, four social attributes in the Oh et al. (2020) dataset, four social attributes in the Oosterhof and Todorov (2008) dataset, and three social attributes in the White et al. (2017) dataset.

To assess how well a model fitted for a social attribute would predict other social attributes (i.e., the last section of Results, “Non-specific Predictions Across Social Attributes”), we did not require ratings on the exact same or highly (dis)similar social attribute between the training dataset and test datasets. Therefore, we fit a model for each of the 14 social attributes in the Chicago Face Database, and assessed how well the predicted ratings from these models correlated with the ratings in the test datasets on all available social attributes (except for the Lin et al. (2021)﻿ dataset, where ratings were measured for 100 social attributes; we only used a subset of 15 social attributes that are commonly studied in the literature).

### DCNN-Identity Features

To extract identity features from face images, we used the dlib ﻿C +  + machine learning library, which offers an open source implementation of face recognition with deep neural networks (King, 2009, 2017). The network’s final layer represents each face image with a vector of 128 features. The network had been originally trained to identify 7,485 face identities in a dataset of about three million faces with a loss function such that the two face images of the same identity were mapped closer to each other in the face space than the face images of two different identities. Built on a ResNet architecture with 29 convolutional layers, the network achieved an accuracy of 99.38% on the “Labeled Faces in the Wild” benchmark (King, 2009, 2017). We directly used the feature vectors from the last layer of the network, without tuning the network or its last layer specifically for social judgments from faces.

### DCNN-Object Features

To extract object features from face images, we used the features obtained from the block5_conv2 layer of the VGG16 network because prior studies showed that features from this layer of the network successfully predicted social judgments from faces (Song et al., 2017). We also repeated our analyses with features from other layers of the network, which produced worse performance (Fig. S1); we therefore used the features from the block5_conv2 layer for subsequent analyses. To extract the object features from a face image, the face region of the image was first detected and segmented automatically using the histogram of oriented gradients-based face detector implemented in the dlib ﻿C + + library (King, 2009, 2017). Then the segmented image was presented to the VGG16 model implemented in the Keras deep learning library (Chollet, 2015) with weights pre-trained on the ImageNet dataset (Deng et al., 2009) for object recognition. The output of the block5_conv2 layer had a volume shape of 14 × 14 × 512, which was flattened into a 100,352-dimensional feature vector. Thus, the layer represented each face image with a vector of 100,352 features.

Due to the large number of features, we used principal component analysis (PCA) to reduce the dimensionality and redundancy of these features. Our goal was to retain a much smaller number of PCs from the 100,352 features, and project the 100,352 features of the face images in both the training dataset and test datasets onto these PCs — which we eventually used in the linear regression models. To prevent biasing the PCs of the faces in the test datasets with the variance in the faces from the training dataset, we performed PCA using a larger and more comprehensive set of faces: face images of 426 white adults with neutral expression aggregated from three popular publicly available face databases (Chelnokova et al., 2014; DeBruine & Jones, 2017; Ma et al., 2015). We determined the optimal number of PCs based on their performance for predicting social judgments from the faces in the model training dataset (i.e., the 183 studio portraits from the Chicago Face Database). Specifically, the 426 faces were first represented with the 100,352-dimensional *DCNN-Object* feature vectors, on which we performed PCA to extract PCs of the features. Next, the 100,352-dimensional feature vectors of the 183 faces in the training dataset were projected onto these PCs obtained from the 426 faces. Finally, we fit ridge regression models using different numbers of PCs (increased from 10 to 110 with a step size of one) to predict the ratings of the 183 faces. Results showed that the first 26 PCs offered the best average prediction accuracy across all 14 social attributes, and we therefore used the first 26 PCs to represent the *DCNN-Object* features in all subsequent analyses (Fig. S1).

### Facial-Geometry Features

The brute-force approach offered by DCNNs has the well-known effect of producing representations, such as the face features described above, that are not easily interpretable. We therefore also used a complementary human-specified set of interpretable face features. The physical and geometric features of the face (e.g., brighter skin, larger eyes, and rounder face) have been shown to influence how humans make social judgments of unfamiliar others based on faces (Ma et al., 2015). To obtain these features, we referred to the 40 facial-geometry features provided in the Chicago Face Database (Ma et al., 2015), which were defined based on a review of the social perception literature (Blair & Judd, 2011; Zebrowitz & Collins, 1997). In the Chicago Face Database, these 40 physical and geometric features were manually measured using an image editing software (Ma et al., 2015). In our present study, given the large number of faces we used, we aimed to generate a subset of those physical and geometric features that could be automatically measured, but were still easily interpretable. A recent study showed that automatically measured physical and geometric features are highly correlated with those that are manually measured (Jones et al., 2021). Here, to automatically measure physical and geometric features, we used a pre-trained model of facial landmark detection implemented in the dlib C + +﻿ library to estimate the location of 68 key points on each face image. This model had been originally built using an ensemble-of-regression-trees approach and trained on the IBUG 300-W facial landmark dataset (Kazemi & Sullivan, 2014; King, 2017; Sagonas et al., 2016). We used another pre-trained model of face parsing to segment each face image into several facial parts such as skin area, left and right eye, and the nose (see Fig. S2). This model has been originally built using a BiSeNet architecture and trained on CelebAMask-HQ dataset (Lee et al., 2020; Yu et al., 2018; Zllrunning, 2020). These automated methods allowed us to obtain 30 physical and geometric features (*Facial-Geometry* features) that closely imitate the manually measured physical and geometric features provided in the Chicago Face Database. The 30 *Facial-Geometry* features were the median luminance of skin area, nose width, nose length, lip thickness, face length, eye height (left, right), eye width (left, right), face width at cheek, face width at mouth, distance between pupils, distance between pupil and upper lip (left, right, asymmetry), chin length, length of cheek to chin (left, right), face shape, (face) heartshapeness, nose shape, lip fullness, eye shape, eye size, midface length, chin size, cheekbone height, cheekbone prominence, face roundness, and facial width-to-height ratio. We verified that the 30 automatically extracted *Facial-Geometry* features described the social judgments from faces as well as the 40 manually measured features by comparing the prediction accuracy of the models based on the two sets of features (see Fig. S2).

### Model Fitting

L2-regularized linear regression (a.k.a. ridge regression; Hoerl & Kennard, 1970) was used to fit a set of model weights separately for each social attribute that optimally mapped facial features onto human subjects’ social judgments from faces (Fig. 1). Cross-validation was used to determine the optimal regularization parameter for ridge regression. Specifically, the training dataset was randomly split into 80% training and 20% validation samples for 2,000 iterations. At each iteration, a range of regularization parameters (*n* = 30, log-spaced between 1 and 100,000) were used to fit models to the training part, and each fitted model was used to predict the human ratings of the faces in the validation part. This procedure yielded a model accuracy per regularization parameter per iteration per social attribute, assessed with the mean squared error (MSE). For each social attribute, the optimal regularization parameter that minimized the average error across all iterations was selected, and the model weights were refit with this optimal regularization parameter using the entire training dataset (i.e., the final model). We also repeated this procedure of selecting regularization parameter using evaluation metrics in addition to MSE, including the coefficient of determination (*R*^2^) and the root mean square error (RMSE) — results corroborated those using MSE reported here.

The final fitted model for each social attribute was used to predict ratings of the same social attribute for the novel faces in each test dataset. Some out-of-sample test datasets did not include ratings of the exact same social attributes as in the training set (i.e., the Chicago Face Database). In those cases, we used the final model for a social attribute (e.g., dominant) to predict ratings of a semantically highly (dis)similar social attribute in the test dataset (e.g., submissive) if that was available. A bootstrap procedure was used to robustly estimate the prediction accuracy of each model on each test dataset. Specifically, the face images and their ratings in each test dataset were randomly sampled 10,000 times with replacement, and the Spearman rank-order correlation between the resampled predicted and resampled human ratings was computed per social attribute (Lescroart & Gallant, 2019). We used the Spearman rank-order correlation to assess model accuracy because the ratings in some test datasets were collected on a different scale than the training dataset and the rank order of faces based on an attribute (i.e., whether a face looks more trustworthy than another face) is a more reliable metric than raw rating values attributed to the faces. The mean prediction accuracy for each social attribute was obtained by averaging the accuracies across bootstrap iterations. For the test dataset that contained a large number of ambient photos (504 photos of 42 white individuals; White et al., 2017), one image was randomly sampled from the set of images available for each identity at each bootstrap iteration (i.e., 42 images were included at each iteration) to prevent bias in prediction accuracy.

To assess the statistical significance of the mean prediction accuracy and estimate the chance threshold for the prediction per social attribute in each test dataset, we performed a permutation analysis to generate an empirical null distribution of correlations for each social attribute and test dataset separately. At each permutation iteration, the ratings in a test dataset were shuffled across face images, and the Spearman correlation between the predicted and permuted ratings was computed for each social attribute. This procedure was repeated 10,000 times to obtain a distribution of the correlations, under the null hypothesis that there is no relationship between facial features and social judgments from faces. The chance threshold was determined by taking the 95th percentile of the empirical null distribution (*p* = 0.05). The permutation *p*-value for each social attribute was defined as the proportion of the null correlations that were greater than or equal to the observed prediction accuracy. The *p*-values were corrected for multiple comparisons across the predicted social attributes using the false discovery rate (FDR) procedure (Benjamini & Hochberg, 1995).

In order to characterize the robustness of our findings to the specific analysis pipeline, we also repeated the above analysis procedures using linear regression methods in addition to ridge regression, including LASSO regression and ordinary least square regression (OLS). The same cross-validation procedure as described for ridge regression was used to select the optimal regularization parameter for LASSO regression from a range of regularization parameters (*n* = 30, log-spaced between 0.01 and 100). No cross-validation procedure was used in training the OLS models since there was no regularization parameter to be determined for this method. We found that ridge regression provided the best predictions across social attributes and test datasets (mean prediction accuracy measured with Spearman’s $\rho$ = 0.552 ± 0.197 for the *DCNN-Identity* models; 0.430 ± 0.218 for the *DCNN-Object* models; 0.385 ± 0.213 for the *Facial-Geometry* models; mean ± standard deviation across test datasets and attributions). In comparison, LASSO regression provided similar prediction accuracies as ridge regression across social attributes and test datasets (mean Spearman’s $\rho$ = 0.527 ± 0.198 for the *DCNN-Identity* models; 0.426 ± 0.226 for the *DCNN-Object* models; 0.369 ± 0.224 for the *Facial-Geometry* models). However, OLS regression provided worse prediction accuracies for the *DCNN-Identity* models (mean Spearman’s $\rho$ = 0.239 ± 0.140 across social attributes and test datasets) and the *Facial-Geometry* models (0.169 ± 0.098) due to multicollinearity in the features, and similar prediction accuracies for the *DCNN-Object* models (0.434 ± 0.221). Therefore, we used the linear regression method that produced the best prediction accuracies across feature spaces, ridge regression, in our present investigation.

### Variance Partitioning Analysis

We used a variance partitioning analysis procedure to compare the unique and shared explained variance between each pair of feature spaces (Çukur et al., 2016; Lescroart & Gallant, 2019). Specifically, for each social attribute and each pair of feature spaces, we fit three models using the training dataset: one fit the ratings to a feature space (e.g., 128 *DCNN-Identity* features), the second fit the ratings to a second feature space (e.g., 26 *DCNN-Object* features), and the third fit the ratings to both feature spaces (e.g., 154 *DCNN-Identity* and *DCNN-Object* features). These three fitted models were used to predict the ratings of the faces in the test dataset. The variance explained (*R*^2^) by each model for each social attribute was computed by using *R*^2^, the coefficient of determination. Finally, the unique variance explained by each of the two compared feature spaces (A and B) and the shared variance explained by both feature spaces were computed as follows:

$${R}_{uA}^{2}={R}_{A\cup B}^{2}-{R}_{B}^{2}$$

$${R}_{uB}^{2}={R}_{A\cup B}^{2}-{R}_{A}^{2}$$

$${R}_{A\cap B}^{2}={R}_{A}^{2}+{R}_{B}^{2}-{R}_{A\cup B}^{2}$$

where ${R}_{A}^{2}$ is the total variance explained by the first model using feature space A, ${R}_{B}^{2}$ is the total variance explained by the second model using feature space B, ${R}_{A\cup B}^{2}$ is the total variance explained by the third model using features from both spaces, ${R}_{uA}^{2}$ is the unique variance explained by feature space A, ${R}_{uB}^{2}$ is the unique variance explained by feature space B, and ${R}_{A\cap B}^{2}$ is the shared variance explained by feature spaces A and B.

### Semi-partial Correlation Analysis

To understand how different social judgments contribute to the cross-predictions across multiple social attributes (i.e., when a model fitted to one social attribute predicted other social attributes), we performed a semi-partial correlation analysis. This analysis procedure measures the relationship between two variables *X* and *Y* while statistically controlling for (or partialing out) the effect of a third variable *Z* on *Y*. Note that, in contrast, the (standard) partial correlation controls for the effect of *Z* on both *X* and *Y*. In this analysis, the actual ratings of a social attribute provided by the human subjects in the test dataset were used as the variable *X* (i.e., the social attribute to be cross-predicted by a model that was not fitted to this social attribute). The ratings of a second social attribute predicted by a model for the same set of faces were used as the variable *Y* (i.e., a second social attribute that was used to fit a model). The ratings of a third social attribute predicted by another model for the same set of faces were used as the variable *Z* (i.e., a third social attribute that was used to fit another model). To partial out the effect of *Z* from *Y*, a simple bivariate regression of *Y* on *Z* was performed, and the residuals were obtained. These residuals quantified the unique variance in *Y* that was not linearly associated with or predictable from Z. Finally, we computed the Spearman correlation coefficient between *X* and the residuals.

## Results

### Generalizability Across Faces, Raters, and Social Attributes

For each social attribute and each facial feature space, we fit a ridge regression model with cross-validation to learn the relationship between the features and human judgments of this social attribute from faces. Results reported here were from models fitted to the popular Chicago Face Database (Ma et al., 2015). We also fit the models using a more recent database that collected ratings from human subjects on a much larger number of social attributes for a representatively sampled set of faces (Lin et al., 2021﻿), whose results corroborated those reported here (see Fig. S3; the models differed in the attributes that they could predict, because the two datasets differed in the social attributes on which human subjects had provided ratings in the first place).

**Fig. 2:**
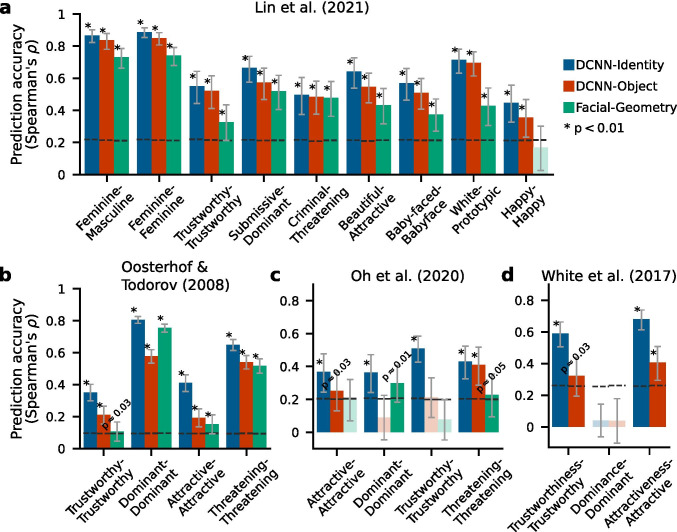
**Prediction accuracy of three feature spaces across different test datasets**. All models were fitted to human subject ratings for 183 studio portraits of frontal, neutral, white faces from the Chicago Face Database (Ma et al., 2015). The *x*-axis indicates the social judgments measured in the test and training datasets (test-training). **a** The prediction accuracy of the models tested on an independent dataset of 60 novel studio portraits of frontal, neutral, white faces and their ratings (Lin et al., 2021﻿). The bar height indicates the mean prediction and error bars indicate the standard deviations of the mean prediction accuracy across bootstrap samples (*n* = 10,000). Saturated colors, asterisks, and *p*-values indicate statistically significant predictions (*p* < 0.05, assessed with permutation tests, and FDR corrected); desaturated colors indicate nonsignificant predictions. Dashed black lines indicate the chance threshold for the prediction accuracy (*p* = 0.05, assessed with permutation test). **b** The prediction accuracy of the models tested on a different independent dataset of 300 computer-generated white faces and their ratings (Oosterhof & Todorov, 2008). **c** The prediction accuracy of the models tested on a different independent dataset of 66 studio portraits of frontal, neutral, white faces and their ratings (Oh et al., 2020). **d** The prediction accuracy of the models tested on a different independent dataset of 504 ambient photos of white faces in the wild (varied in viewpoint, facial expression, background, illumination, etc.; White et al., 2017) and their ratings (42 images were used in each bootstrap iteration, see “2”). The automatic extraction of Facial-Geometry features was not feasible for these faces.

To investigate how well the predictions of these linear regression models with different feature spaces generalized across faces, raters, and social attributes, we tested the models on multiple out-of-sample datasets. These out-of-sample datasets consisted of ratings on various social attributes from independent sets of human subjects for faces that were different from those used in the training set. While the training stimuli were all drawn from studio portraits, the out-of-sample datasets encompassed studio portraits as well as ambient photos (taken in real-world contexts) that varied in viewpoint, facial expression, and background. Since the social attributes with available human ratings in the test datasets were not always identical to those in the training dataset, we only computed the prediction accuracy for social attributes in the test datasets that were the same or semantically highly similar (or the exact opposite) to those in the training dataset (e.g., predicting *submissive* ratings in the test dataset using the models fitted to the *dominant* ratings in the training dataset by multiplying the model weights with -1﻿). Results summarized in Fig. 2 showed that the *DCNN-Identity* models significantly predicted judgments of almost all social attributes across all datasets (except *dominant* ratings for ambient photos, Fig. 2d), and yielded a higher prediction accuracy across social attributes and test datasets (Spearman’s correlations 0.55 ± 0.19, mean ± SD across all social attributes and test datasets) than the *DCNN-Object* models (0.43 ± 0.22) or the *Facial-Geometry* models (0.38 ± 0.21). We also explicitly examined the generalization across raters only (using a subset of overlapping faces in the training and one test dataset) and found similar relative performances across the three feature spaces (Fig. S4). These results demonstrate that, out of our three feature spaces, features from the pre-trained DCNN for face identification provided the best generalizable predictions across faces, raters, and social attributes.

The superior performance of the *DCNN-Identity* models over the *DCNN-Object* and *Facial-Geometry* models raises two questions. First, is this superior performance simply due to the much larger number of features in the *DCNN-Identity* models (*n* = 128)? Second, is this superior performance idiosyncratic to the specific network used to derive those *DCNN-Identity* features? To address the first question, we applied principal component analysis (PCA) on the *DCNN-Identity* features and used only the first 30 PCs for fitting the models, a number close to the number of features in the *DCNN-Object* (*n* = 26) and the *Facial-Geometry* models (*n* = 30). To address the second question, we fit the models using features from a different DCNN for face identification that has an architecture distinct from the *DCNN-Identity* network, the OpenFace DCNN (Amos et al., 2016). The performance of the *DCNN-Identity* PC models was as good as with the original *DCNN-Identity* models, and the superior performance of the original *DCNN-Identity* models was not idiosyncratic to the specific network architecture (Fig. S5).

### Comparison Across Feature Spaces

We have shown that models using *DCNN-Identity* features predicted social judgments from faces at a higher accuracy than models using the other two feature spaces across various social attributes and test datasets. We next sought to quantify the variance explained by models using each of these three feature spaces. We performed a variance partitioning analysis (see “2; Çukur et al., 2016; Lescroart & Gallant, 2019) to identify the proportion of variance in the social judgments that was uniquely explained by each feature space and the proportion of variance that was commonly explained by any two feature spaces. Models were fitted to the Chicago Face Database and tested using the Lin et al. (2021) dataset (as in Fig. 2a).

**Fig. 3:**
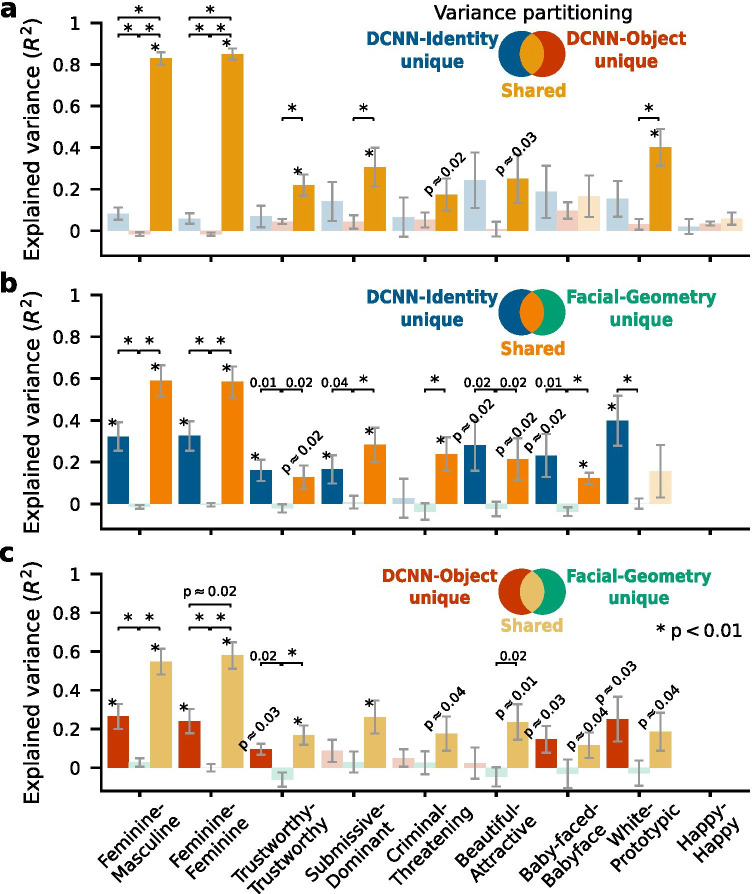
**Results of variance partitioning analyses**. All models were fitted to the Chicago Face Database and tested on the Lin et al. (2021) dataset as in Fig. 2a. **a** Variance partitioning between the DCNN-Identity and DCNN-Object models. Error bars show bootstrap standard deviations of the explained variance across bootstrap samples (*n* = 10,000 iterations). Saturated colors, and the asterisks and *p*-values next to the error bars indicate that the explained variance was significantly different from zero (*p* < 0.05, assessed with bootstrap tests, FDR corrected). Desaturated colors indicate that the explained variance was not significantly different from zero. The asterisks and *p*-values above the horizontal brackets indicate statistically significant differences in the explained variance between unique and shared components (*p* < 0.05, bootstrap tests, FDR corrected). **b** Variance partitioning between the DCNN-Identity and Facial-Geometry models (this analysis was not performed for the attribute “happy” because the Facial-Geometry model failed to predict this judgment in the test dataset, see Fig. 2a). **c** Variance partitioning between the DCNN-Object and Facial-Geometry models

The variance partitioning analysis revealed that the *DCNN-Identity* and *DCNN-Object* models accounted for almost the same variance in the test datasets (Fig. 3a). The *Facial-Geometry* model, on the other hand, was not able to explain any unique variance beyond that shared with the other two feature spaces (Fig. 3b–c). These findings indicate that most of the variance in the social judgments that was explained by any of the three feature spaces could be explained by the *DCNN-Identity* feature space alone.

**Fig. 4:**
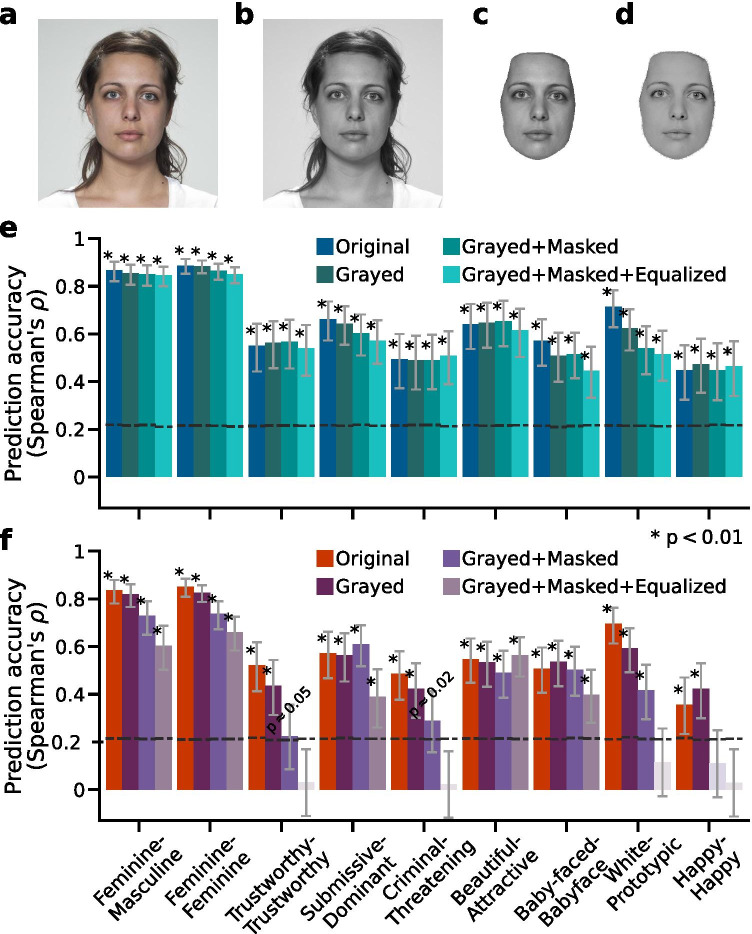
**Prediction accuracy as a function of low-level image properties**. Models were fitted to the Chicago Face Database and tested on the Lin et al. (2021) dataset as in Fig. 2a. **a** An example of a face image before any manipulation. **b** An example of the face image in (**a**) manipulated on colors (i.e., converted to gray-scale). **c** An example of the face image in (**a**) manipulated on hair style (i.e., hair was removed). **d** An example of the face image in (**a**) manipulated on mean luminance (i.e., the face area luminance histograms were equalized across cropped gray-scale face images in the test dataset). **e** The accuracy of using the model weights obtained from the training dataset (unmanipulated version of the faces) and the *DCNN-Identity* features extracted from the manipulated versions (**a**–**d**) of the faces in the test dataset to predict the human subject ratings of the unmanipulated version of the faces in the same test dataset. **f** Same as (**e**) except that the features were *DCNN-Object* features

The highly similar explained variance between the *DCNN-Identity* and the *DCNN-Object* feature spaces raises an interesting question: do the two feature spaces provide equally robust predictions? To provide insights into this question, we manipulated the face images in the test dataset on a set of low-level image properties—their color, hair region, and mean luminance (Fig. 4). We expected these changes to have minimal impact on how humans make social judgments from the faces. We used the previously fitted regression model weights (i.e., models fitted to the unmanipulated version of the faces as in Fig. 2a) and the features of the manipulated versions of the face images in the test dataset (extracted using the *DCNN-Identity* network and the *DCNN-Object* network, respectively) to predict the human ratings of the unmanipulated version of the face images.

We found that the manipulation of these low-level image properties yielded a larger decline in the prediction accuracy of the *DCNN-Object* models (mean accuracy difference $\Delta \rho$ = 0.28 across social attributes, especially in the predictions of *trustworthy*, *criminal*, *white*, and *happy*), but only a slight drop in the prediction accuracy of the *DCNN-Identity* model ($\Delta \rho$ = 0.05 across social attributes) as shown in Fig. 4e–f. These results indicate that the *DCNN-Identity* features carry face-specific information (e.g., identity and gender) that is largely robust to the changes in image styles (Hill et al., 2019; O’Toole et al., 2018). In contrast, the *DCNN-Object* features carry substantial information about image-based characteristics (e.g., illumination and hair parts close to face area), limiting the generalizability of predicting human social judgments of the same face in different image styles.

Taken together, these results indicate that DCNNs pre-trained to recognize face identity produce features that can be used most successfully to predict social judgments made by humans from faces. These predictions generalize well across faces, raters, social attributes, and image styles. However, features from the DCNN pre-trained to recognize objects and the physical and geometric features were less robust and generalizable for predicting social judgments from faces.

### Non-specific Predictions Across Social Attributes

Having identified the best performing feature space (the *DCNN-Identity* features), we next sought to understand whether the predictions made by our regression models were based on a specific pattern of weights for each social attribute. Considerable prior work has shown that the hundreds of different words people use to describe judgments of others from faces could be represented by just a few psychological dimensions (typically 2–4 dimensions account for > 70% of the variance in ratings; Jones & Kramer, 2021; Lin et al., 2021﻿; Oosterhof & Todorov, 2008; Sutherland et al., 2013). These findings highlight the possibility that the individual models fitted for different social attributes would also be correlated (Todorov et al., 2013, 2015). Indeed, we found that social judgments that were correlated in the original human subject ratings across face images were also correlated in their model weights across features (Fig. S6). None of the correlations computed with the human subject ratings was significantly different from the correlation computed with the estimated model weights for the same pair of social attributes (bootstrap tests, *p* > 0.05, FDR corrected).

These results raise a concern that the models fitted to predict a certain social judgment might in fact learn the representation of other correlated social judgments. Therefore, we investigated to which degree a model fitted to predict a certain social attribute would also predict the judgments of other social attributes regarding the same face (“cross-prediction”). We assessed the cross-prediction accuracy with the Spearman correlation between the ratings predicted by the model for a certain social attribute (e.g., *feminine*) and the ratings collected from human subjects for a different social attribute (e.g., *criminal*) regarding the same set of faces in the test dataset. All analyses in this section used the same training dataset and test datasets as in Fig. 2 (with one additional test dataset; Walker et al., 2018).

**Fig. 5:**
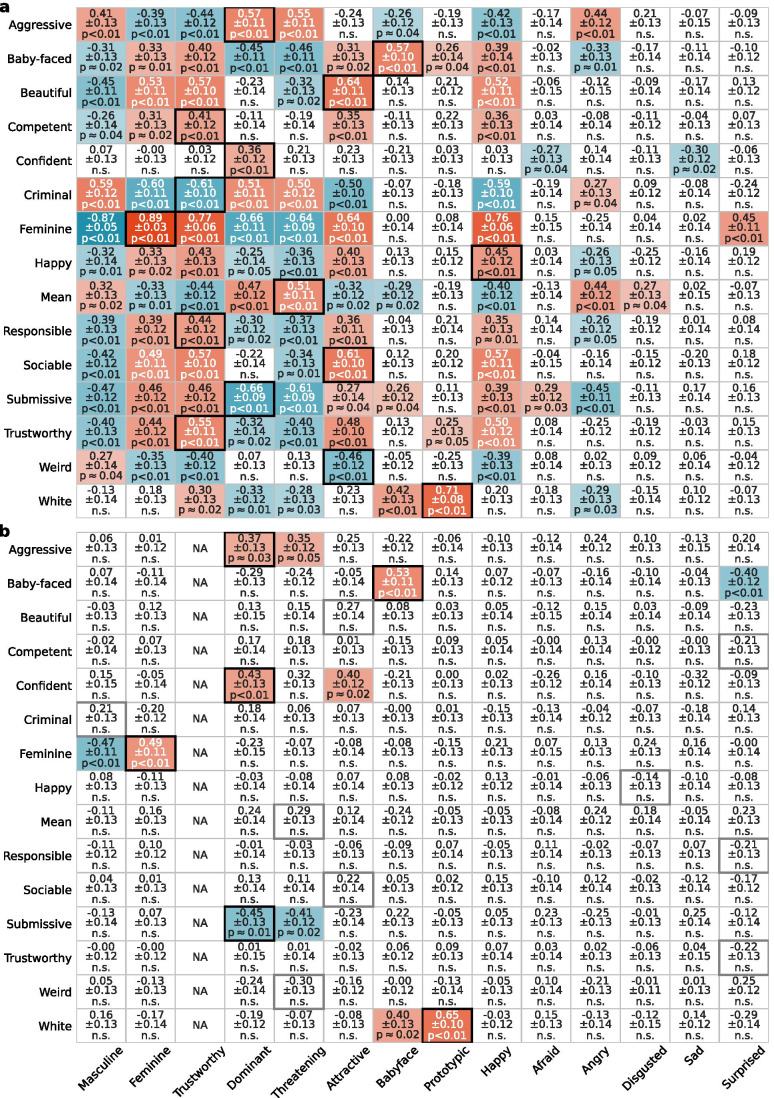
**Cross-prediction accuracy across social attributes**. **a** Cross-prediction accuracy (the Spearman correlations) between the predicted ratings of the faces in the Lin et al. (2021)﻿ dataset on 14 social attributes (*x*-axis) and the human subject ratings of the same set of faces on 15 social attributes available in the test dataset (*y*-axis). All social attribute models (*x*-axis) were fitted to the Chicago Face Database. Statistically significant accuracy values are colored. The saturation of the color indicates the magnitude of the correlation (red for positive, blue for negative). Numbers indicate the mean and standard deviation (across bootstrap samples), and the significance of the correlation (permutation test, FDR corrected). The highest accuracy per row was highlighted with a solid box (black for significant, gray for nonsignificant). **b** An example of the residual cross-prediction accuracy for the social attributes in the Lin et al. (2021)﻿ test dataset (*y*-axis) from 13 social attribute models (*x*-axis) while controlling for the prediction from the trustworthy model (the third column; shown here specifically because for this test dataset, the trustworthy model had the largest impacts on cross-predictions across all 14 models fitted to all 14 social attributes in the Chicago Face Database). Numbers report the mean bootstrap residual cross-prediction accuracy, bootstrap standard deviation, and significance level computed via permutation tests and FDR corrected (*n* = 10,000 iterations)

We found significant cross-predictions across social attributes in all test datasets (Fig. 5a, Figs. S7a, S8a, S9a, c, e). For instance, the model fitted to predict *feminine* judgments from faces not only predicted *feminine* judgments in the test dataset as intended (Figs. 5a and 2a) but also predicted how much human subjects judged the faces in the test dataset to be *aggressive*, *baby-faced*, *beautiful*, *competent*, *criminal*, *happy*, etc*.* (Fig. 5a). What is even more concerning is that some models (e.g., *trustworthy* in Fig. 5a) predicted a different social judgment (e.g., *feminine* and *criminal*) at a higher accuracy than the social judgment that they were fitted to and intended to predict (*trustworthy*) (see also Fig. S7a and Fig. S8a). These results indicate that automated predictions of human social judgments from faces are not attribute-specific.

**Fig. 6:**
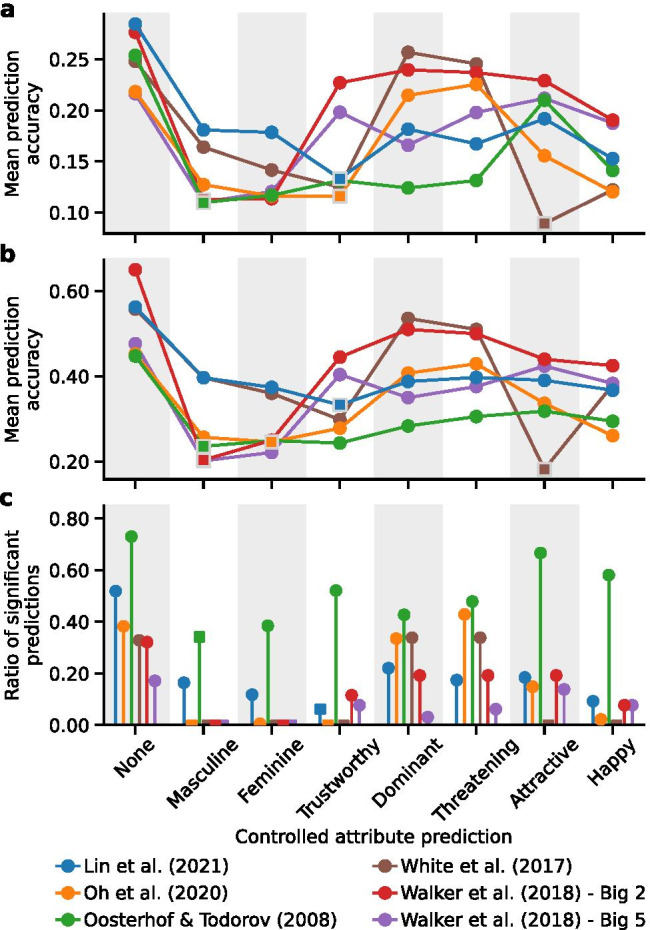
**Residual cross-prediction accuracy after partialing out the effect of another social attribute model**. **a** The first column (“None”) plots the mean cross-prediction accuracy (dots; i.e., mean absolute Spearman’s correlations) across all cross-predictions in each test dataset (all cells in Fig. 5a, Figs. S7a, S8a, S9a, c, e). The other columns plot the mean residual cross-prediction accuracy across all cross-predictions after partialing out the effect of the model fitted for the social attributes labeled in the *x*-axis. The top seven (of the 14) social attributes that had the largest effect on cross-predictions across test datasets are shown here. The square symbol (rather than filled circle) indicates the social attribute model (*x*-axis) that was the most impactful for cross-predictions in a test dataset (i.e., minimum mean residual cross-prediction accuracy). **b** The first column (“None”) plots the mean maximum cross-prediction accuracy (the absolute maximum correlation per row in Fig. 5a, Figs. S7a, S8a, S9a, c, e) across all social attributes in a test dataset (*y*-axis in Fig. 5a, Figs. S7a, S8a, S9a, c, e). The other columns plot the mean maximum residual cross-prediction accuracy across all social attributes in a test dataset after partialing out the effect of the social attribute model labeled in the *x*-axis. **c** The first column (“None”) plots the ratio of significant cross-predictions across all cross-predictions in each test dataset. The other columns plot the ratio of significant cross-predictions after partialing out the effect of the social attribute model labeled in the *x*-axis

Given these cross-predictions (e.g., Fig. 5a), we next investigated whether there are some social judgments that play a more important role in leading to the cross-predictions than others. For each cross-prediction (e.g., using the *feminine* model to predict ratings of *criminal* in the test dataset), we computed the *residual* cross-prediction accuracy after partialing out the effect of each remaining social attribute model (i.e., the 13 social attribute models in the *x*-axis of Fig. 5a except for *feminine*). The *residual* cross-prediction accuracy (Fig. 5b, Figs. S7b, S8b, S9b, d, f) was assessed with the semi-partial Spearman’s correlation between the human ratings of a social attribute for the faces in a test dataset (e.g., *criminal*) and the residuals from a simple bivariate regression of the predicted ratings of a different social attribute from a model (e.g., *feminine* model) on the predicted ratings of a third social attribute from a remaining model (e.g., *trustworthy* model) for the faces in the same test dataset (these residuals quantify the unique variance in the predicted *feminine* ratings that were not associated with the predicted *trustworthy* ratings). Figure 6 summarizes the mean *residual* cross-prediction accuracy in each test dataset after partialing out the effect of each remaining model corresponding to each social attribute in the *x*-axis. We found that models predicting gender (*masculine*/*feminine*) played a more important role for cross-prediction of personality traits from faces (the “Big-2” and “Big-5” personality dimensions) and social judgments of computer-generated faces (Oosterhof & Todorov, 2008; Walker et al., 2018). Models predicting *trustworthy* played a more important role in test datasets where the photos were neutral and taken for research purposes (Lin et al., 2021﻿; Oh et al., 2020). The model predicting *attractiveness* was more important for ambient social media profile photos (White et al., 2017).

## Discussion

In this paper, we examined the generalizability, robustness, and specificity of a recent popular modeling approach for automatically predicting social judgments made by human perceivers from faces. This approach trained regularized linear regression models (ridge regression with cross-validation) to predict social judgments from faces using features extracted from the face images based on DCNNs pre-trained for other purposes than social judgments from faces (Fig. 1). We compared the predictive power of these features to that of the physical and geometric features of the faces, which are traditionally studied in psychology research. We tested these regularized linear regression models built with different feature sets using five independent out-of-sample test datasets, which included ratings from different human participants, for various types of faces, and on a range of social and affective attributes (Fig. 2).

We found that regression models built with features from DCNNs that were pre-trained to distinguish facial identity (*DCNN-Identity*) predicted human judgments from faces most accurately and generalized the best across faces and raters (Fig. 2 and Fig. S4), compared to the models built with DCNN features for object recognition (*DCNN-Object*) or features based on facial geometry (*Facial-Geometry*). The performance of the *DCNN-Identity* models was robust to the dataset used to fit the models (Fig. S3), the number of features included in the regression models (Fig. S5), and the network architecture used to obtain the identity features (Fig. S5).

Using variance partitioning analysis, we showed that the *DCNN-Identity* models and the *DCNN-Object* models explain almost the same variance in the social judgments from faces (Fig. 3). However, the features extracted by the two DCNNs from face images that were relevant for predicting social judgments from the faces differed in prediction robustness. Features extracted using the pre-trained *DCNN-Identity* network represented more information unique to the faces (e.g., identity and gender), whereas features extracted using the pre-trained *DCNN-Object* network represented more information about the images in general (e.g., color and parts of the images), and thus the predictions from the *DCNN-Object* network features were less robust to manipulations of image styles (Fig. 4).

Although the *DCNN-Identity* features provided the most accurate, robust, and generalizable predictions of social and affective judgments from faces, we note that these predictions were not attribute-specific. Models fitted to predict judgments of a certain social attribute from faces also predicted judgments of other unintended social attributes (Fig. 5a, Figs. S7a, S8a, S9a, c, e). Some models even predicted other unintended social attributes at a greater accuracy than the intended social attributes that the models were fitted to (Fig. 5a, Fig. S7a, and Fig. S8a). These results indicate that the representation of social judgments from faces that the models learned might not be specific to the social judgment that the researchers intend to predict. This finding is an important cautionary note: one should be aware that there are likely many other correlated but unintended social judgments that might explain the predictions.

In this cross-prediction analysis, we also included models fitted to six affective attributes (*happy*, *afraid*, *angry*, *disgusted*, *sad*, *surprised*) since this analysis did not require the same or highly (dis)similar social attributes to be available in the out-of-sample test datasets. We examined how these affective models predicted judgments of other social attributes from the faces (all with emotionally neutral expressions). Prior research has shown that people’s social judgments from emotionally neutral faces are influenced by the face’s structural resemblance to emotional expressions (e.g., individuals whose emotionally neutral face images look like they are somewhat angry are judged to be more aggressive; Said et al., 2009). In line with this research, we found that models fitted to affective judgments of the faces significantly cross-predicted other social judgments (e.g., Fig. 5). However, the number of other social judgments that these affective models cross-predicted was on average much smaller than the models fitted to other social attributes (*attractive*, *baby-faced*, *dominant*, *feminine*, *masculine*, *prototypic*, *threatening*, *trustworthy*), likely because the faces used in our study were all intended to be emotionally neutral. The non-affective social attributes in our study describe more temporally stable characteristics of a person, and therefore their judgments from faces are more likely to be linked to the structural features of the face, which were captured by the features from DCNNs pre-trained for face identification. Since the judgments of affective attributes from the face are likely to be shaped by more temporally changeable features of the face (e.g., facial expression) that are more difficult to be captured by the features from DCNNs pre-trained for face identification, this results in worse prediction (e.g., *happy* model in Fig. 2) as well as fewer cross-predictions (e.g., Fig. 5). Future research directions could address these issues by combining features from DCNNs pre-trained for face identification and for emotion categorization, and using faces with strong expressions (ideally several from the same person).

Finally, we provided a novel analysis, semi-partial correlation analysis, for understanding how different social attributes contribute to the cross-predictions across social judgments (e.g., Fig. 5a). We found that the most important social attribute for cross-predictions varied depending on the context. For instance, the judgments of *trustworthy* seem to play a more important role in test datasets consisting of neutral face photos taken for research purposes, whereas the judgment of *attractiveness* was more important in the test dataset where the photos were taken for social media profiles by the users themselves (Fig. 6). The social attributes we examined in this analysis were constrained by the social attributes whose ratings were available in the training dataset. With datasets that include a more comprehensive set of social attributes in future studies, our approach could be applied to these broader social attributes to help understand the most important social judgments from faces for human perceivers in different contexts (e.g., photos taken for different purposes, or for different types of decision-making).

Several limitations of our study constrain the generality of our findings. First, the most important limitation is that the rating data we used to fit and test our models almost certainly lack validity. That is, even though there is considerable consensus in the social judgments made by humans from faces (generating the “ground-truth” labels for training our models; Rule et al., 2010), the majority of those judgments do not reflect the actual attribute of the person whose face is shown. Instead, those judgments mainly reveal our biases and stereotypes (Sutherland et al., 2020; Todorov, 2017). This limitation is even more acute given that all stimuli in both the training and test datasets were isolated faces devoid of context and any other information about the person. Our results thus show that it is possible to predict what people judge or believe about brief glances of a face, but not what is in fact valid about the person whose face is shown as the stimulus. Needless to say, it is critical to keep this distinction in mind: we did not predict anything about the people whose faces were used; instead, we predicted what human viewers judge about those faces.

Our conclusions were also limited by the small number of overlapping social attributes between the training dataset and the different test datasets, and thus the small number of social attributes for which we could construct meaningful models. Finally, we only included white faces in our analysis, since these were by far the predominant race available in the training datasets, and since there are known and important race bias effects. Previous work has shown that social judgments from faces are influenced by the unique facial features of faces from different races as well as the different social concepts associated with the different races (Fan et al., 2020; Stolier & Freeman, 2016). Altogether, the restricted range of different types of faces and the small number of social attributes provide results that are not yet comprehensive. Analyses on future datasets that are more complete in the social judgments and more diverse in the face stimuli will be valuable to extend the present study.

We conclude with two remarks. First, our analysis pipeline could be flexibly applied to other domains of automated predictions to better understand their generalizability, robustness, and underlying mechanisms. This analysis pipeline includes testing models using multiple independent out-of-sample datasets, performing variance partitioning analysis to compare between models, manipulating stimulus properties to test robustness, and conducting cross-prediction analysis to examine potential correlated predictions. Second, given that the many social judgments humans make from faces are highly correlated, future research attempting to automatically predict social judgments from faces (e.g., predicting whether people judge a face to look criminal) or even the actual characteristics of the people whose faces are used as stimuli (e.g., predicting who is criminal from their face) should be cautious when interpreting those predictions. Specifically, we would recommend that researchers examine other potentially correlated social judgments before drawing conclusions.

## Supplementary Information

Below is the link to the electronic supplementary material.

Supplementary file1 (DOCX 4.03 MB)

## Additional Information

### Data Availability

All data are from publicly available datasets which could be accessed via the links provided in the papers cited.

### Code Availability

All analysis codes are available at https://osf.io/xdsvp/?view_only=6efb50f8b6bd49a493ac9c64ddf630e6.

## References

[CR1] Ahler DJ, Citrin J, Dougal MC, Lenz GS (2017). Face value? Experimental evidence that candidate appearance influences electoral choice. Political Behavior.

[CR2] Amos B, Ludwiczuk B, Satyanarayanan M (2016). OpenFace: A general-purpose face recognition library with mobile applications. CMU School of Computer Science.

[CR3] Bainbridge WA, Isola P, Oliva A (2013). The intrinsic memorability of face photographs. Journal of Experimental Psychology: General.

[CR4] Barrett LF, Adolphs R, Marsella S, Martinez AM, Pollak SD (2019). Emotional expressions reconsidered: Challenges to inferring emotion from human facial movements. Psychological Science in the Public Interest.

[CR5] Benjamini Y, Hochberg Y (1995). Controlling the false discovery rate: A practical and powerful approach to multiple testing. Journal of the Royal Statistical Society. Series B (Methodological).

[CR6] Blair IV, Judd CM (2011). Afrocentric facial features and stereotyping. The Science of Social Vision.

[CR7] Bowyer KW, King MC, Scheirer WJ, Vangara K (2020). The “Criminality From Face” illusion. IEEE Transactions on Technology and Society.

[CR8] Chelnokova O, Laeng B, Eikemo M, Riegels J, Løseth G, Maurud H, Leknes S (2014). Rewards of beauty: The opioid system mediates social motivation in humans. Molecular Psychiatry.

[CR9] Chollet, F., & others. (2015). Keras [Github]. Retrieved June 10, 2021, from https://keras.io

[CR10] Çukur T, Huth AG, Nishimoto S, Gallant JL (2016). Functional subdomains within scene-selective cortex: Parahippocampal place area, retrosplenial complex, and occipital place area. The Journal of Neuroscience.

[CR11] D’Amour, A., Heller, K., Moldovan, D., Adlam, B., Alipanahi, B., Beutel, A., … Sculley, D. (2020). Underspecification presents challenges for credibility in modern machine learning. ArXiv:2011.03395 [Cs, Stat]. Retrieved from http://arxiv.org/abs/2011.03395

[CR12] DeBruine, L., & Jones, B. (2017). Face Research Lab London Set.10.6084/m9.figshare.5047666.v3

[CR13] Deng, J., Dong, W., Socher, R., Li, L., Kai Li, & Li Fei-Fei. (2009). ImageNet: A large-scale hierarchical image database. 2009 IEEE Conference on Computer Vision and Pattern Recognition, 248–25510.1109/CVPR.2009.5206848

[CR14] Engell AD, Haxby JV, Todorov A (2007). Implicit trustworthiness decisions: Automatic coding of face properties in the human amygdala. Journal of Cognitive Neuroscience.

[CR15] Fan X, Wang F, Shao H, Zhang P, He S (2020). The bottom-up and top-down processing of faces in the human occipitotemporal cortex. ELife.

[CR16] Gheorghiu AI, Callan MJ, Skylark WJ (2017). Facial appearance affects science communication. Proceedings of the National Academy of Sciences.

[CR17] Hamermesh DS (2011). *Beauty pays: Why attractive people are more successful*.

[CR18] Hill MQ, Parde CJ, Castillo CD, Colón YI, Ranjan R, Chen J-C, O’Toole AJ (2019). Deep convolutional neural networks in the face of caricature. Nature Machine Intelligence.

[CR19] Hoerl AE, Kennard RW (1970). Ridge regression: Biased estimation for nonorthogonal problems. Technometrics.

[CR20] Jones AL, Kramer RSS (2021). Facial first impressions form two clusters representing approach-avoidance. Cognitive Psychology.

[CR21] Jones AL, Schild C, Jones BC (2021). Facial metrics generated from manually and automatically placed image landmarks are highly correlated. Evolution and Human Behavior.

[CR22] Kazemi V, Sullivan J (2014). One millisecond face alignment with an ensemble of regression trees. IEEE Conference on Computer Vision and Pattern Recognition.

[CR23] King DE (2009). Dlib-ml: A machine learning toolkit. Journal of Machine Learning Research.

[CR24] King, D. E. (2017). Dlib-models [Github]. Retrieved June 10, 2021, from https://github.com/davisking/dlib-models

[CR25] Lee, C.-H., Liu, Z., Wu, L., & Luo, P. (2020). MaskGAN: Towards diverse and interactive facial image manipulation. 2020 IEEE/CVF Conference on Computer Vision and Pattern Recognition (CVPR), 5548–5557. Seattle, WA, USA: IEEE. 10.1109/CVPR42600.2020.00559

[CR26] Lenz GS, Lawson C (2011). Looking the part: Television leads less informed citizens to vote based on candidates’ appearance. American Journal of Political Science.

[CR27] Lescroart MD, Gallant JL (2019). Human scene-selective areas represent 3D configurations of surfaces. Neuron.

[CR28] Lewenberg, Y., Bachrach, Y., Shankar, S., & Criminisi, A. (2017). Predicting personal traits from facial images using convolutional neural networks augmented with facial landmark information. Proceedings of the Thirtieth AAAI Conference on Artificial Intelligence, 4365–4366.

[CR29] Lin C, Adolphs R, Alvarez RM (2017). Cultural effects on the association between election outcomes and face-based trait inferences. PLoS ONE.

[CR30] Lin, C., Keles, U., & Adolphs, R. (2021). Four dimensions characterize attributions from faces using a representative set of English trait words. *Nature Communications*. 12, 5168. 10.1038/s41467-021-25500-y 10.1038/s41467-021-25500-y PMC8397784 34453054

[CR31] Ma DS, Correll J, Wittenbrink B (2015). The Chicago face database: A free stimulus set of faces and norming data. Behavior Research Methods.

[CR32] Martin DS (1978). Person perception and real-life electoral behaviour. Australian Journal of Psychology.

[CR33] McCurrie M, Beletti F, Parzianello L, Westendorp A, Anthony S, Scheirer WJ (2018). Convolutional neural networks for subjective face attributes. Image and Vision Computing.

[CR34] Oh, D., Dotsch, R., Porter, J., & Todorov, A. (2020). Gender biases in impressions from faces: Empirical studies and computational models. *Journal of Experimental Psychology: General*. 149(2), 323–342. 10.1037/xge0000638 10.1037/xge0000638 31294585

[CR35] Oldmeadow J, Sutherland C, Young A (2013). Facial stereotype visualization through image averaging. Social Psychological and Personality Science.

[CR36] Oliviola, C., Eastwick, P., Finkel, E., Hortaçu, A., Ariely, D., & Todorov, A. (2015). First impressions and consumer mate preferences in online dating and speed-dating. *ACR North American Advances*, *43*. Retrieved from https://www.acrwebsite.org/volumes/1019800/volumes/v43/NA-43

[CR37] Oosterhof NN, Todorov A (2008). The functional basis of face evaluation. Proceedings of the National Academy of Sciences.

[CR38] O’Toole AJ, Castillo CD, Parde CJ, Hill MQ, Chellappa R (2018). Face space representations in deep convolutional neural networks. Trends in Cognitive Sciences.

[CR39] O’Toole AJ, Harms J, Snow SL, Hurst DR, Pappas MR, Ayyad JH, Abdi H (2005). A video database of moving faces and people. IEEE Transactions on Pattern Analysis and Machine Intelligence.

[CR40] Parde, C. J., Hu, Y., Castillo, C., Sankaranarayanan, S., & O’Toole, A. J. (2019). Social trait information in deep convolutional neural networks trained for face identification. *Cognitive Science*, *43*(6). 10.1111/cogs.12729 10.1111/cogs.12729 PMC8735867 31204800

[CR41] Rule NO, Ambady N, Adams RB, Ozono H, Nakashima S, Yoshikawa S, Watabe M (2010). Polling the face: Prediction and consensus across cultures. Journal of Personality and Social Psychology.

[CR42] Sagonas C, Antonakos E, Tzimiropoulos G, Zafeiriou S, Pantic M (2016). 300 Faces in-the-wild challenge: Database and results. Image and Vision Computing.

[CR43] Said, C. P., Sebe, N., & Todorov, A. (2009). Structural resemblance to emotional expressions predicts evaluation of emotionally neutral faces. *Emotion*. 9(2), 260–264. 10.1037/a0014681 10.1037/a0014681 19348537

[CR44] Simonyan, K., & Zisserman, A. (2015). Very deep convolutional networks for large-scale image recognition. ArXiv:1409 1556 [Cs]. Retrieved from https://arxiv.org/abs/1409.1556

[CR45] Song A, Linjie L, Atalla C, Cottrell G (2017). Learning to see faces like humans: Modeling the social dimensions of faces. Journal of Vision.

[CR46] Stolier RM, Freeman JB (2016). Functional and temporal considerations for top-down influences in social perception. Psychological Inquiry.

[CR47] Sutherland CAM, Burton NS, Wilmer JB, Blokland GAM, Germine L, Palermo R, Rhodes G (2020). Individual differences in trust evaluations are shaped mostly by environments, not genes. Proceedings of the National Academy of Sciences.

[CR48] Sutherland CAM, Liu X, Zhang L, Chu Y, Oldmeadow JA, Young AW (2018). Facial first impressions across culture: Data-driven modeling of Chinese and British perceivers’ unconstrained facial impressions. Personality and Social Psychology Bulletin.

[CR49] Sutherland CAM, Oldmeadow JA, Santos IM, Towler J, Michael Burt D, Young AW (2013). Social inferences from faces: Ambient images generate a three-dimensional model. Cognition.

[CR50] Todorov A (2017). *Face value: The irresistible influence of first impressions*.

[CR51] Todorov A, Dotsch R, Porter JM, Oosterhof NN, Falvello VB (2013). Validation of data-driven computational models of social perception of faces. Emotion.

[CR52] Todorov A, Mandisodza AN, Goren A, Hall CC (2005). Inferences of competence from faces predict election outcomes. Science.

[CR53] Todorov A, Olivola CY, Dotsch R, Mende-Siedlecki P (2015). Social attributions from faces: Determinants, consequences, accuracy, and functional significance. Annual Review of Psychology.

[CR54] Walker M, Schönborn S, Greifeneder R, Vetter T (2018). The Basel Face Database: A validated set of photographs reflecting systematic differences in Big Two and Big Five personality dimensions. PLoS ONE.

[CR55] Wang Y, Kosinski M (2018). Deep neural networks are more accurate than humans at detecting sexual orientation from facial images. Journal of Personality and Social Psychology.

[CR56] White D, Sutherland CAM, Burton AL (2017). Choosing face: The curse of self in profile image selection. Cognitive Research: Principles and Implications.

[CR57] Willis J, Todorov A (2006). First impressions: Making up your mind after a 100-ms exposure to a face. Psychological Science.

[CR58] Wilson JP, Rule NO (2015). Facial trustworthiness predicts extreme criminal-sentencing outcomes. Psychological Science.

[CR59] Yu C, Wang J, Peng C, Gao C, Yu G, Sang N, Ferrari V, Hebert M, Sminchisescu C, Weiss Y (2018). BiSeNet: Bilateral segmentation network for real-time semantic segmentation. Computer Vision – ECCV 2018.

[CR60] Zebrowitz, L. A., & Collins, M. A. (1997). Accurate social perception at zero acquaintance: The affordances of a Gibsonian approach. *Personality and Social Psychology Review: An Official Journal of the Society for Personality and Social Psychology, Inc*, *1*(3), 204–223. 10.1207/s15327957pspr0103_2 10.1207/s15327957pspr0103_2 15659350

[CR61] Zebrowitz LA, Voinescu L, Collins MA (1996). “Wide-eyed” and “crooked-faced”: Determinants of perceived and real honesty across the life span. Personality and Social Psychology Bulletin.

[CR62] Zllrunning. (2020). Face-parsing.PyTorch [Github]. Retrieved June 10, 2021, from https://github.com/zllrunning/face-parsing.PyTorch

