# Supplementary material for: A Cautionary Note on Predicting Social Judgments from Faces with Deep Neural Networks
Source: Affect Sci. 2021 Sep 20;2(4):438–54. doi: 10.1007/s42761-021-00075-5 (PMC8664800; doi:10.1007/s42761-021-00075-5)
Supplement: Supplementary file 1 — Supplementary file1 (DOCX 4.03 MB) [file 42761_2021_75_MOESM1_ESM.docx]

**Electronic supplementary material**

**A cautionary note on predicting social judgments from faces with deep neural networks**

Umit Keles^*,1^, Chujun Lin^2^, Ralph Adolphs^1,3^

^1^Division of the Humanities and Social Sciences, California Institute of Technology, Pasadena, CA, USA.

^2^Department of Psychological and Brain Sciences, Dartmouth College, Hanover, NH, USA.

^3^Division of Biology and Biological Engineering, California Institute of Technology, Pasadena, CA, USA.

^*^Correspondence to: [ukeles@caltech.edu](mailto:umit@caltech.edu)

**Supplementary Figures**


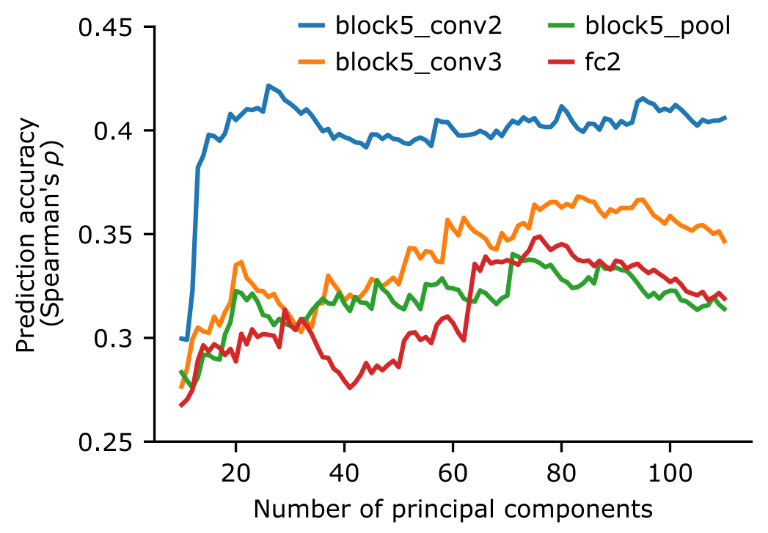


**Fig. S1** Prediction accuracy made using features obtained from different layers of the VGG16 neural network, including the block5_conv2, block5_conv3, block5_pool, and fc2 layers, and different number of principal components (PCs). The same model fitting procedure was used for all layers and different number of PCs. Specifically, for each different layer of the VGG16 network and the number of PCs, the data (i.e., the 183 faces and their ratings) were randomly split into 80% training set and 20% test set over 2,000 iterations. At each iteration, the outer-loop training data were further randomly split into 80% inner-loop training set and 20% validation set for 20 times to select an optimal regularization parameter for ridge regression separately for each social attribute. After selecting an optimal regularization parameter at each outer-loop iteration per social attribute, the models were refit to outer-loop training set and tested on the outer-loop held-out test set. Line plots show the mean prediction accuracy across all outer-loop cross-validation iterations (n = 2000) and all 14 social attributes. The best prediction performance was achieved using the 26 features obtained from the block5_conv2 layer.

**
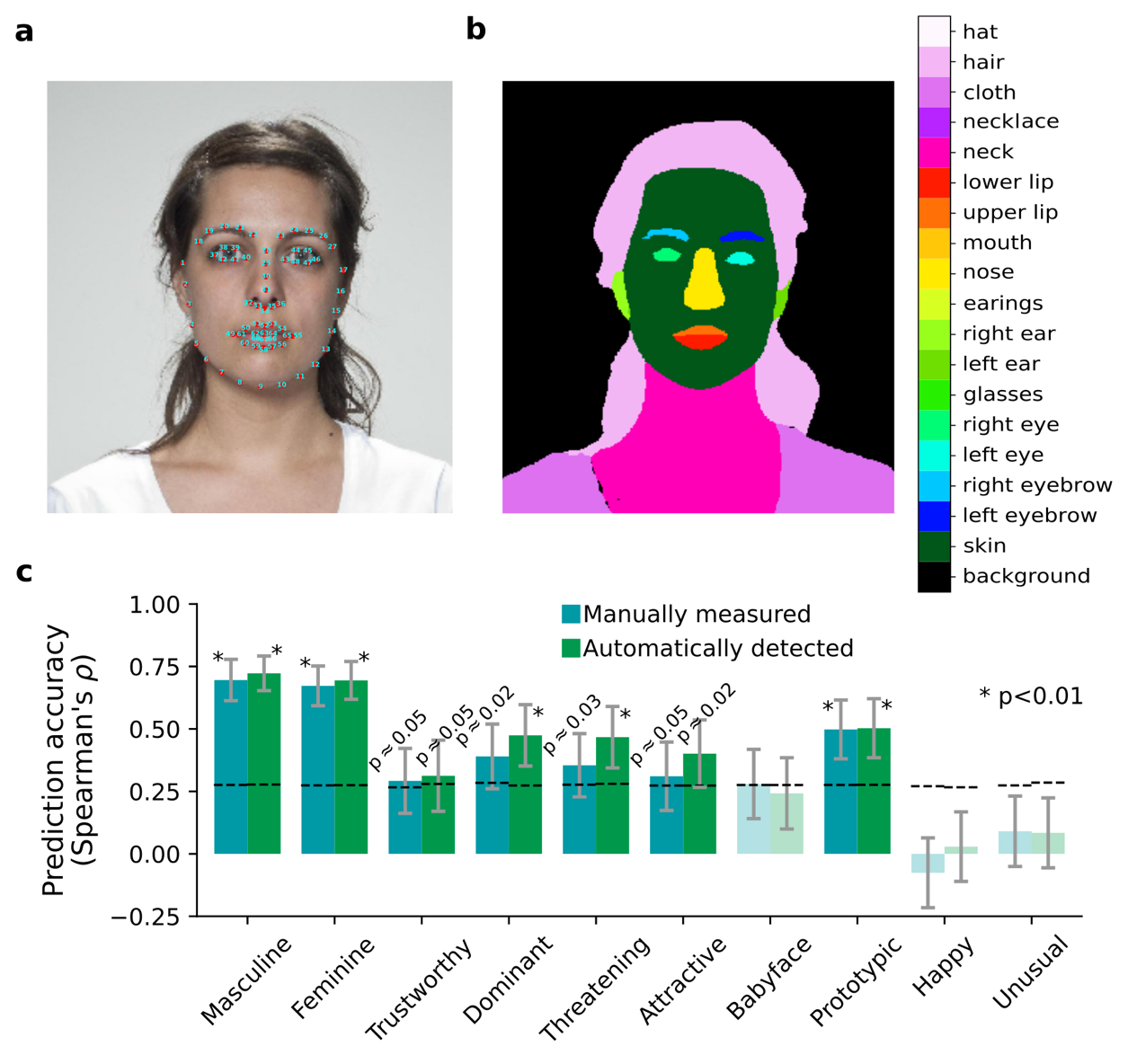
**

**Fig. S2** Automatically extracted Facial-Geometry features. **a,** Automatic detection of facial landmark points. **b,** Automatic detection of facial parts. These landmark points and facial parts were used to automatically measure 30 facial metrics from face images, referred to as *Facial-Geometry* features (e.g., pupillary distance, eye width, median luminance). **c,** Comparison of prediction accuracy from models using automatically detected *Facial-Geometry* features to models using manually measured facial features provided in the Chicago Face Database (Ma, Correll, & Wittenbrink, 2015). Models were trained and evaluated on the data from the Chicago Face Database using a nested cross-validation procedure. For this, the data were randomly split into 80% training set and 20% test set over 2,000 iterations. At each iteration, the outer training data were further randomly split into 80% inner-loop training set and 20% validation set for 20 times to select an optimal regularization parameter for ridge regression. After selecting an optimal regularization parameter at each outer-loop iteration per social attribute, the models were refit to outer-loop training set and tested on the outer-loop held-out test set. The bar height indicates the mean prediction accuracy and error bars indicate the standard deviation of the mean prediction accuracy across outer-loop cross-validation iterations (n = 2000). Saturated colors, asterisks, and p-values indicate statistically significant predictions (*p* < 0.05, assessed with permutation tests, and FDR corrected); desaturated colors indicate nonsignificant predictions. Dashed black lines indicate the chance threshold for the prediction accuracy (*p* = 0.05, assessed with permutation test).


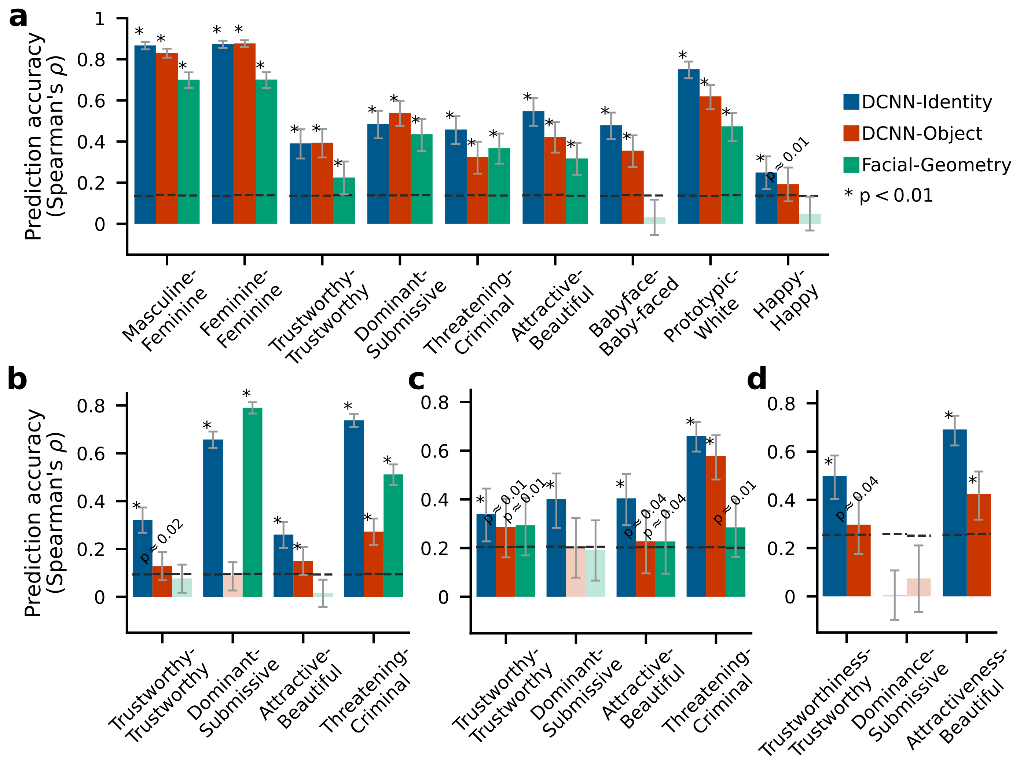


**Fig. S3** Prediction accuracy of models trained on the dataset of (Lin, et al., 2021)**.** All models were trained on a different dataset than the Chicago Face Database (Ma et al., 2015). **a,** The prediction accuracy of models tested on the Chicago Face Database (143 nonoverlapping faces between the Chicago Face Database and the current training dataset were used). The bar height indicates the mean prediction accuracy and error bars indicate the standard deviations of the mean prediction accuracy across bootstrap samples (n = 10,000). Saturated colors, asterisks, and p-values indicate statistically significant predictions (p < 0.05, assessed with permutation tests, and FDR corrected); desaturated colors indicate nonsignificant predictions. Dashed black lines indicate the chance levels (p = 0.05, assessed with permutation test). **b,** The prediction accuracy of the models tested on the test dataset in Fig. 2b with 300 computer-generated white faces and their social attribute ratings (Oosterhof & Todorov, 2008). **c,** The prediction accuracy of the models tested on the test dataset in Fig. 2c with 66 studio portraits (Oh, Dotsch, Porter, & Todorov, 2020). **d,** The prediction accuracy of the models tested on the test dataset in Fig. 2d with 504 ambient photos of faces in the wild (White, Sutherland, & Burton, 2017).


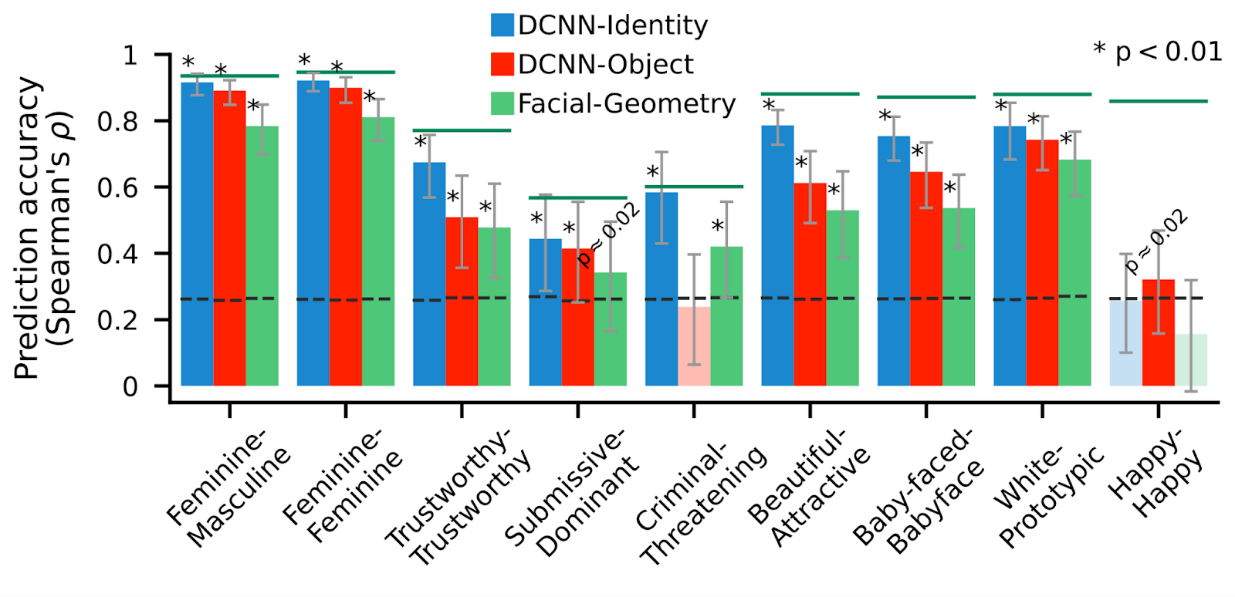


**Fig. S4** Prediction accuracy on Lin et al. (2021) for an overlapping set of faces with the training dataset (n = 40). Different colors indicate the prediction accuracy of different feature spaces. Models were tested on the face images that were available in both the training dataset and the Lin et al. (2021) test dataset, but the ratings were from two independent sets of participants in these two datasets. Green lines show the Spearman correlation between the ratings from the two studies across the faces. These lines indicate the theoretical upper bound for the prediction accuracy of a perfect model. Saturated colors, asterisks, and p-values indicate statistically significant predictions (*p* < 0.05, assessed with permutation tests, and FDR corrected); desaturated colors indicate nonsignificant predictions. Dashed black lines indicate the chance threshold for the prediction accuracy (*p* = 0.05, assessed with permutation test).


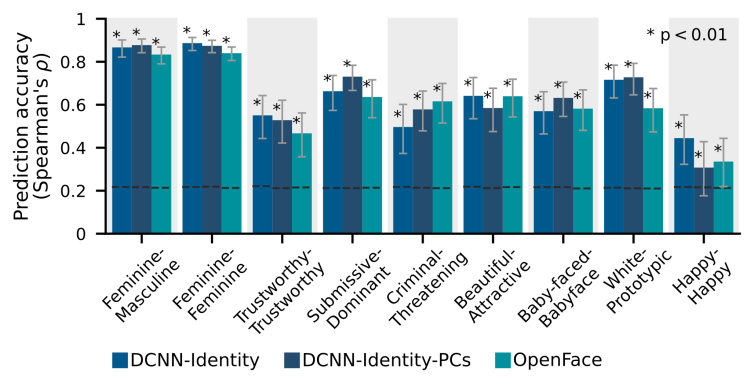


**Fig. S5** Prediction accuracy and the different aspects of the identity features. Prediction accuracy of *DCNN-Identity* models in Fig. 2a (light blue) compared to the prediction accuracy of models that used only 30 principal components of the *DCNN-Identity features* (dark blue)—the same number of regressors as in the *Facial-Geometry* models, and to the prediction accuracy of models using identity features from a different DCNN (turquoise; “OpenFace”; Amos, Ludwiczuk, & Satyanarayanan, 2016).


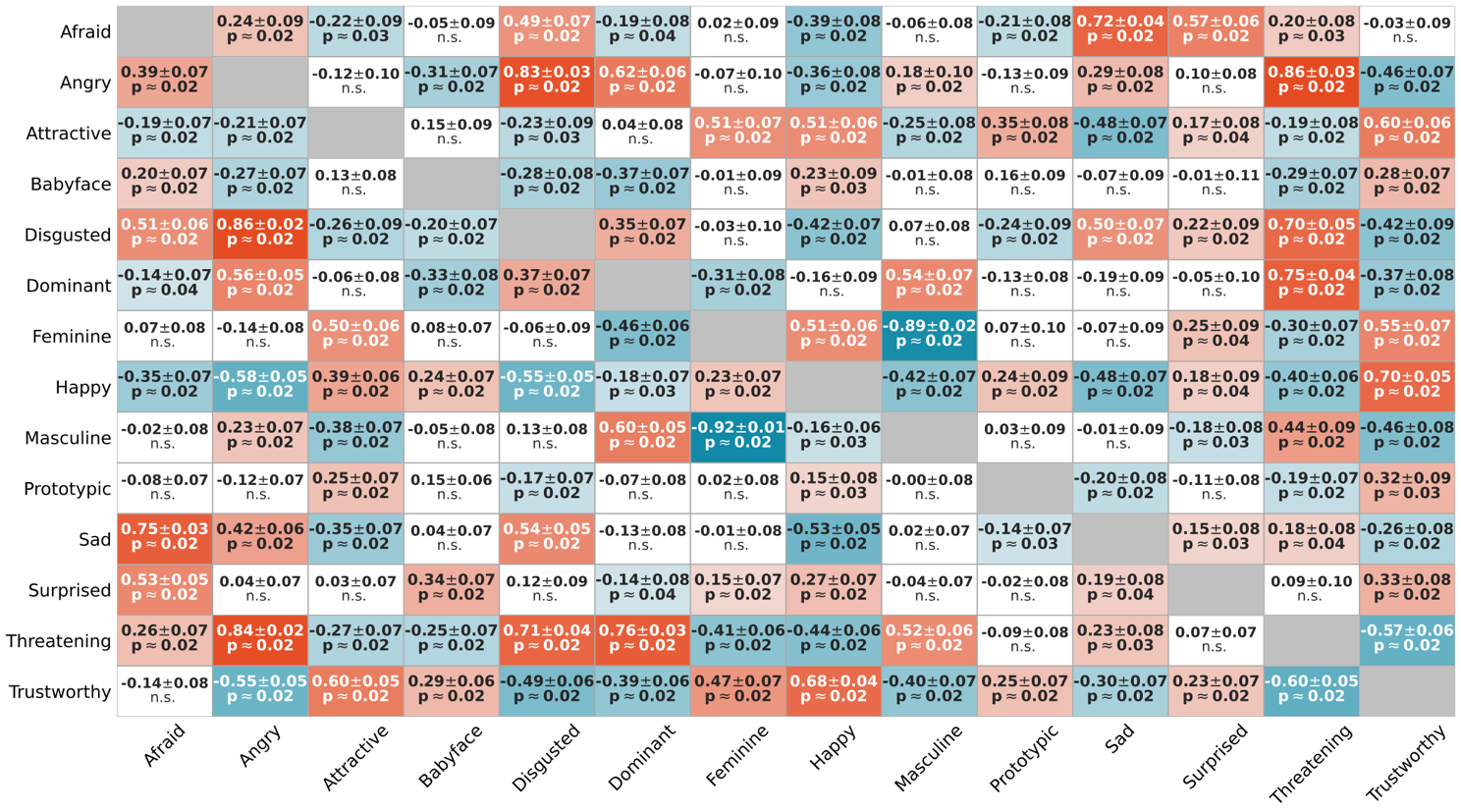


**Fig. S6** Correlations between social attributes in human subject ratings and estimated model weights. The lower-triangle panel shows the Spearman correlations among social attributes computed using the human subject ratings across face images per attribute in the training dataset. The upper-triangle panel shows the Spearman correlations among social attributes computed using the estimated model weights across features per attribute in the training dataset. The saturation of color indicates the magnitude of the correlation (red for positive, blue for negative). Numbers indicate the mean, standard deviation, and significance of the correlation (bootstrap tests, FDR corrected).


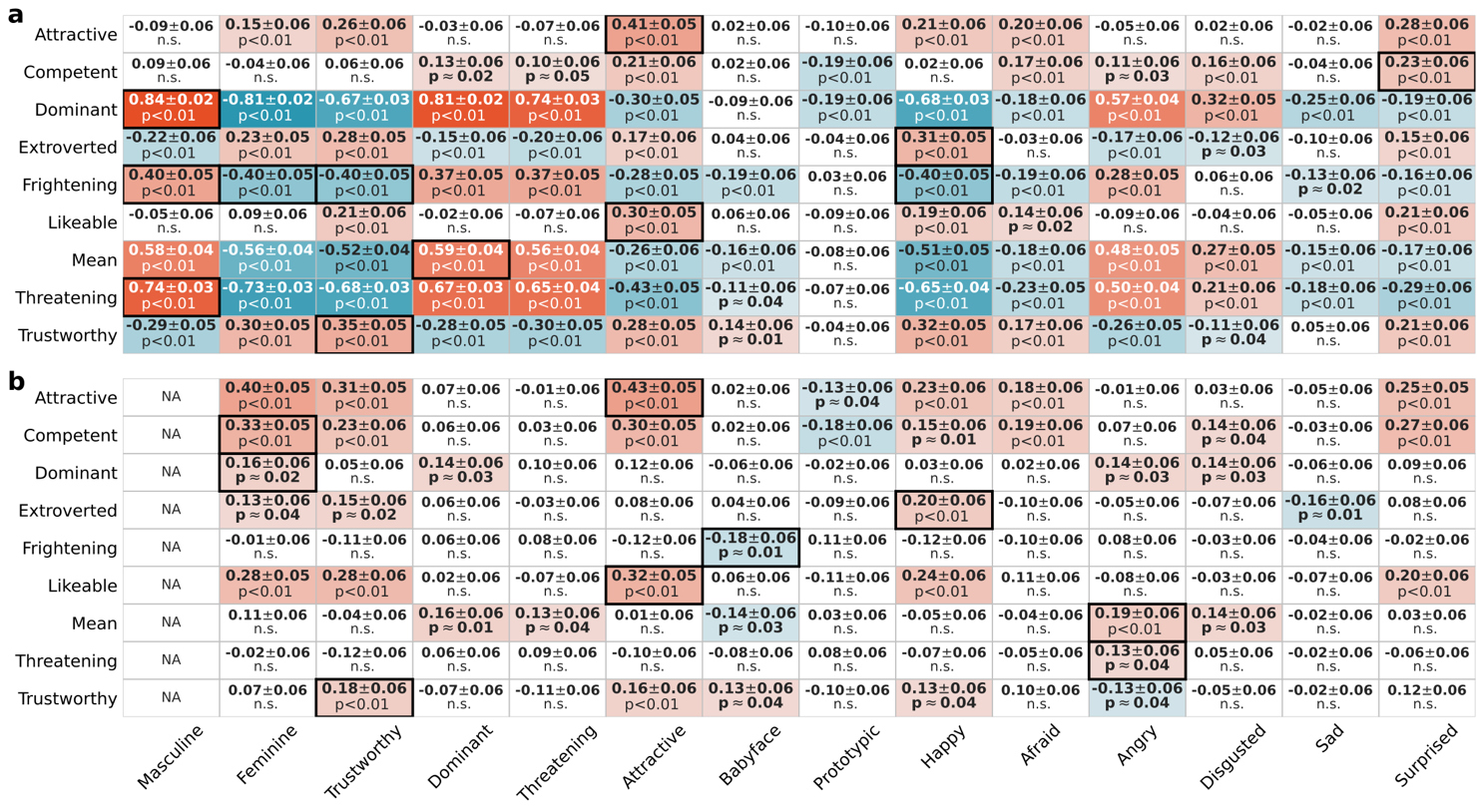


**Fig. S7** Cross-prediction accuracy for the test dataset in Fig. 2b (Oosterhof & Todorov, 2008). **a.** Cross-prediction accuracy (the Spearman correlations) between the predicted ratings of the faces in the test dataset used in Fig. 2b on 14 social attributes(x-axis) and the human subject ratings of the same set of faces on another nine social attributes (y-axis). The saturation of the color indicates the magnitude of the correlation (red for positive, blue for negative). Numbers indicate the mean, standard deviation, and the significance of the correlation (bootstrap tests, FDR corrected). **b.** An example of *residual* cross-prediction accuracy for social attributes in the test dataset used in Fig. 2b (y-axis) from 13 social attribute models (x-axis) while controlling for the prediction from the *masculine* model (selected specifically for this test dataset for its largest impacts on cross-predictions across the 14 social attribute models). Numbers report the mean bootstrap residual cross-prediction accuracy, bootstrap standard deviation, and significant level computed via permutation tests and FDR corrected. The significant accuracy was colored (red for positive, blue for negative; more saturated for greater magnitudes); the highest accuracy per row was highlighted with a solid box (black for significant, grey for nonsignificant).


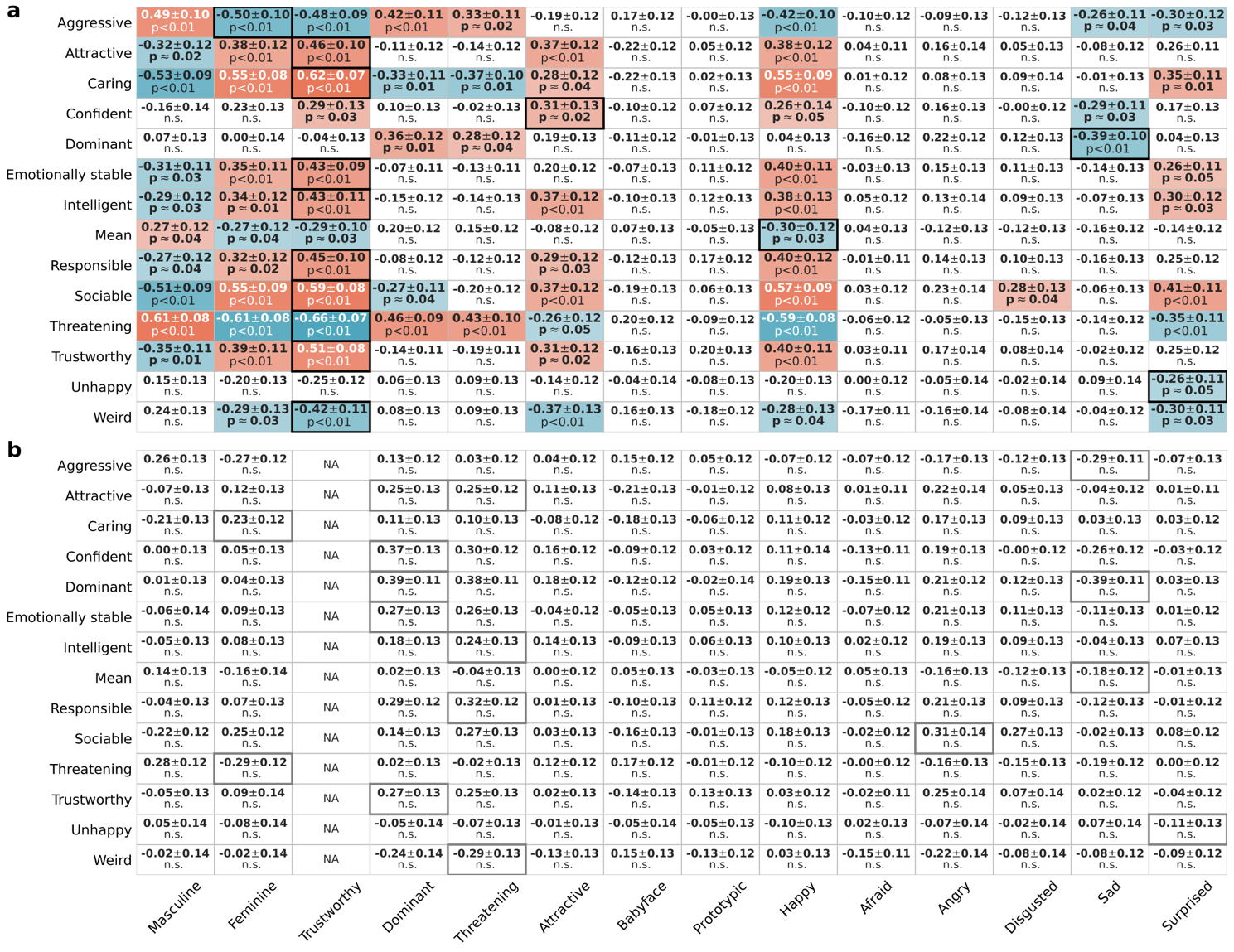


**Fig. S8** Cross-prediction accuracy for the test dataset in Fig. 2c (Oh et al., 2020). **a.** Cross-prediction accuracy (the Spearman correlations) between the predicted ratings of the faces in the test dataset used in Fig. 2c on 14 social attributes (x-axis) and the human subject ratings of the same set of faces on 14 social attributes (y-axis). The saturation of the color indicates the magnitude of the correlation (red for positive, blue for negative). Numbers indicate the mean, standard deviation, and the significance of the correlation (bootstrap tests, FDR corrected). **b.** An example of *residual* cross-prediction accuracy for attributes in the test dataset used in Fig. 2c (y-axis) from 13 social attribute models (x-axis) while controlling for the prediction from the *trustworthy* model (selected specifically for this test dataset for its largest impacts on cross-predictions across the 14 social attribute models). The significant accuracy was colored (red for positive, blue for negative; more saturated for greater magnitudes).


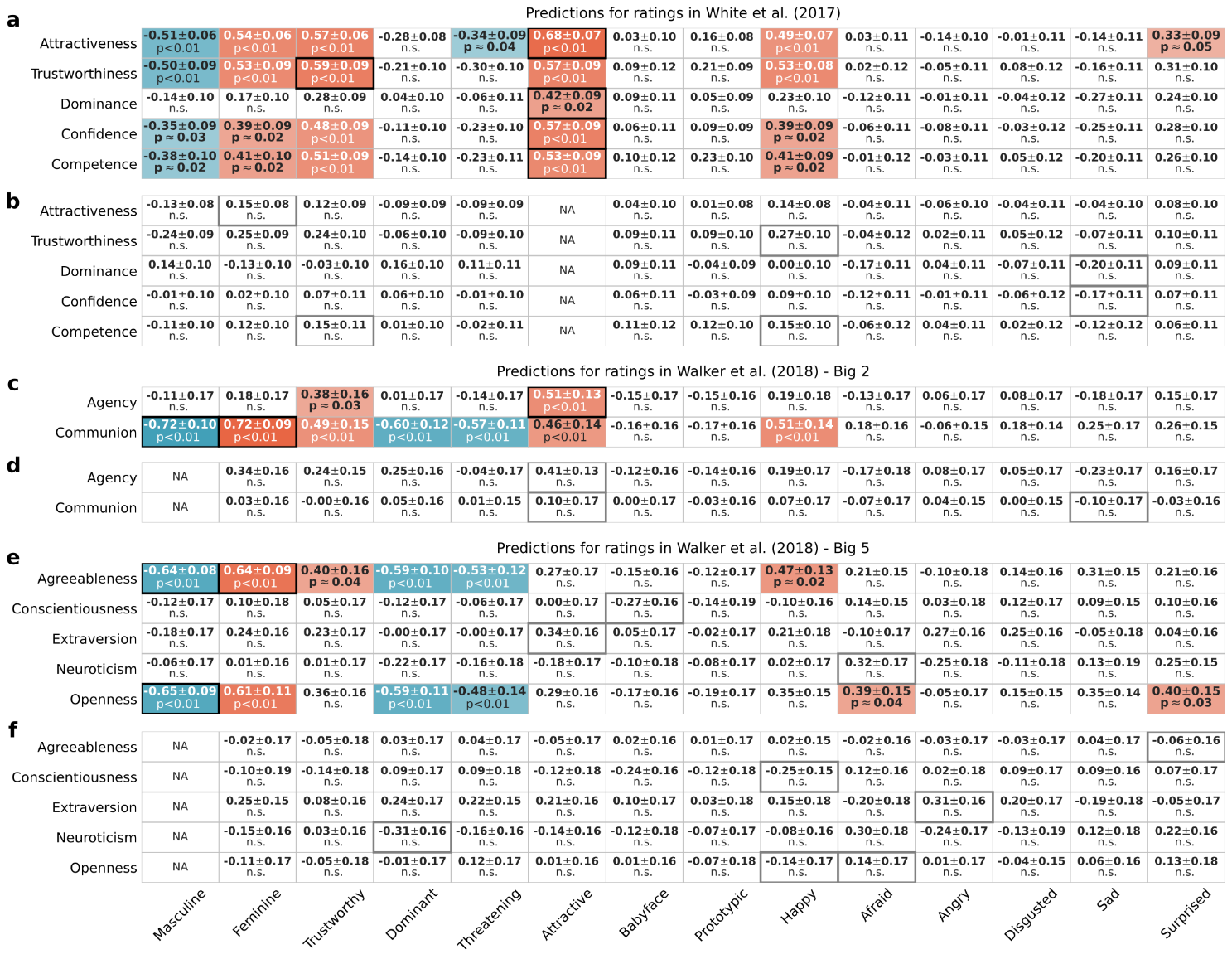


**Fig. S9** Cross-prediction accuracy across three test datasets. **a,** Cross-prediction accuracy (the Spearman correlations) between the predicted ratings of the faces in the test dataset used in Fig. 2d (White et al., 2017) on 14 social attributes (x-axis) and the human subject ratings of the same set of faces on five social attributes (y-axis). **b,** An example of *residual* cross-prediction accuracy for social attributes in the test dataset used in Fig. 2d (y-axis) from 13 social attribute models (x-axis) while controlling for the prediction from the *attractive* model (selected specifically for this test dataset for its largest impacts on cross-predictions across the 14 social attribute models). The significant accuracy was colored (red for positive, blue for negative; more saturated for greater magnitudes). **c,** Cross-prediction accuracy between the predicted ratings of the faces in a fifth out-of-sample test dataset (Walker, Schönborn, Greifeneder, & Vetter, 2018) on 14 attributes (x-axis) and the human subject ratings of the same set of faces on two social attributes (y-axis). **d,** An example of *residual* cross-prediction accuracy for attributes in the test dataset as in (**c**) (y-axis) from 13 social attribute models (x-axis) while controlling for the prediction from the *masculine* model (selected specifically for this test dataset for its largest impacts on cross-predictions across the 14 social attribute models). The significant accuracy was colored (red for positive, blue for negative; more saturated for greater magnitudes). **e,** Cross-prediction accuracy between the predicted ratings of the faces in a sixth out-of-sample test dataset (Walker et al., 2018) on 14 social attributes (x-axis) and the human subject ratings of the same set of faces on five social attributes (y-axis). **f,** An example of *residual* cross-prediction accuracy for social attributes in the test dataset as in (**e**) (y-axis) from 13 attribute models (x-axis) while controlling for the prediction from the *masculine* model (selected specifically for this test dataset for its largest impacts on cross-predictions across the 14 attribute models). The significant accuracy was colored (red for positive, blue for negative; more saturated for greater magnitudes).

**References**

Amos, B., Ludwiczuk, B., & Satyanarayanan, M. (2016). *OpenFace: A general-purpose face recognition library with mobile applications*. 20.

Lin, C., Keles, U., & Adolphs, R. (2021). Four dimensions characterize attributions from faces using a representative set of English trait words. *Nature Communications*, *12*(1), 5168. https://doi.org/10.1038/s41467-021-25500-y

Ma, D. S., Correll, J., & Wittenbrink, B. (2015). The Chicago face database: A free stimulus set of faces and norming data. *Behavior Research Methods*, *47*(4), 1122–1135. https://doi.org/10.3758/s13428-014-0532-5

Oh, D., Dotsch, R., Porter, J., & Todorov, A. (2020). Gender biases in impressions from faces: Empirical studies and computational models. *Journal of Experimental Psychology: General*, *149*(2), 323–342. https://doi.org/10.1037/xge0000638

Oosterhof, N. N., & Todorov, A. (2008). The functional basis of face evaluation. *Proceedings of the National Academy of Sciences*, *105*(32), 11087–11092. https://doi.org/10.1073/pnas.0805664105

Walker, M., Schönborn, S., Greifeneder, R., & Vetter, T. (2018). The Basel Face Database: A validated set of photographs reflecting systematic differences in Big Two and Big Five personality dimensions. *PLOS ONE*, *13*(3), e0193190. https://doi.org/10.1371/journal.pone.0193190

White, D., Sutherland, C. A. M., & Burton, A. L. (2017). Choosing face: The curse of self in profile image selection. *Cognitive Research: Principles and Implications*, *2*(1), 23. https://doi.org/10.1186/s41235-017-0058-3
